# Comparison of Methods for Picking the Operational Taxonomic Units From Amplicon Sequences

**DOI:** 10.3389/fmicb.2021.644012

**Published:** 2021-03-24

**Authors:** Ze-Gang Wei, Xiao-Dan Zhang, Ming Cao, Fei Liu, Yu Qian, Shao-Wu Zhang

**Affiliations:** ^1^Institute of Physics and Optoelectronics Technology, Baoji University of Arts and Sciences, Baoji, China; ^2^Key Laboratory of Information Fusion Technology of Ministry of Education, School of Automation, Northwestern Polytechnical University, Xi’an, China; ^3^Faculty of Electronic and Information Engineering, Xi’an Jiaotong University, Xi’an, China; ^4^School of Mathematics and Statistics, Shaanxi Xueqian Normal University, Xi’an, China

**Keywords:** operational taxonomic units, 16S rRNA, metagenomics, sequence clustering, high-throughput sequencing

## Abstract

With the advent of next-generation sequencing technology, it has become convenient and cost efficient to thoroughly characterize the microbial diversity and taxonomic composition in various environmental samples. Millions of sequencing data can be generated, and how to utilize this enormous sequence resource has become a critical concern for microbial ecologists. One particular challenge is the OTUs (operational taxonomic units) picking in 16S rRNA sequence analysis. Lucky, this challenge can be directly addressed by sequence clustering that attempts to group similar sequences. Therefore, numerous clustering methods have been proposed to help to cluster 16S rRNA sequences into OTUs. However, each method has its clustering mechanism, and different methods produce diverse outputs. Even a slight parameter change for the same method can also generate distinct results, and how to choose an appropriate method has become a challenge for inexperienced users. A lot of time and resources can be wasted in selecting clustering tools and analyzing the clustering results. In this study, we introduced the recent advance of clustering methods for OTUs picking, which mainly focus on three aspects: (i) the principles of existing clustering algorithms, (ii) benchmark dataset construction for OTU picking and evaluation metrics, and (iii) the performance of different methods with various distance thresholds on benchmark datasets. This paper aims to assist biological researchers to select the reasonable clustering methods for analyzing their collected sequences and help algorithm developers to design more efficient sequences clustering methods.

## Introduction

Bacteria constitute an overwhelming majority of domain in the life tree on our planet, occurring in every habitat on earth from natural environments (e.g., oceans, soils, and lakes) to the human body (Sanli et al., 2015; Fuks et al., 2018; Gentile and Weir, 2018). They perform critical functions that range from the regulation of various biogeochemical activities to that of our health and disease (Shah et al., 2018; Thaiss, 2018; Almeida et al., 2019; Qu et al., 2019a). Describing the taxonomic structure of the communities is vital for studying the bacterial composition and diversity in an environmental or clinical sample (Wei et al., 2016; Lapierre et al., 2019; Zhu et al., 2019). Until recently, most of the bacteria were studied with traditional culture-dependent methods. Because only a small fraction (less than 1%) of all microbial organisms can be isolated, cultivated, and identified in the laboratory, culture-dependent microbial methods are inadequate for exploring the hidden world of many microbial communities (Kellenberger, 2001). On the contrary, metagenomics study is a rapidly growing field that aims to understand all organisms via their nucleic acid sequences to characterize the composition, structure, diversity, and function of microbial communities in a specific habitat (Jo, 2004; Riesenfeld et al., 2004; Laudadio et al., 2019; Wemheuer et al., 2020). Bypassing the needs for isolation and lab cultivation of individual species in traditional microbial studies (Streit and Schmitz, 2004; Meyer et al., 2019), metagenomics allows microbiologists to study the entire genetic materials taken directly from relevant environments and provides a new opportunity to probe the microbial community composition and structure (Koslicki et al., 2013; Zhang et al., 2013; Gao, 2018; Wei and Zhang, 2018; Chong et al., 2020; Qian et al., 2020). Thus, several large-scale metagenomics projects, such as the Human Microbiome Project (Turnbaugh et al., 2007; Integrative HMP (iHMP) Research Network Consortium, 2014), the International Census of Marine Microbes^1^, and the Earth Microbiome Project (Gilbert et al., 2014), have been proposed.

In metagenomics, the 16S rRNA (ribosomal RNA) exists in most bacterial species and contains hypervariable regions that allow them to be used as species-specific signatures for identifying taxa (Ward et al., 1990; Stackebrandt and Goebel, 1994; Peterson et al., 2019). Therefore, the 16S rRNA is an ideal proxy for profiling of complex microbial communities and inferring the phylogenetic and evolutionary relations among organisms (Woloszynek et al., 2019). Recently, the rapid advancements in next-generation sequencing (NGS) technologies have dramatically promoted metagenomics studies by offering low-cost and ultra-high-throughput sequencing (Wu et al., 2011). This enormous progress in NGS has resulted in an explosive accumulation of 16S rRNA sequence data (Zhu et al., 2019). How to deal with this massive quantities and high complexity of sequencing data has become a tremendous challenge for microbial researchers (Li et al., 2012; Kim et al., 2013; Qian et al., 2019). As a result, it is needed to develop efficient and accurate computational methods for analyzing these enormous sequence data generated from different habitats and health conditions (Huang et al., 2010; Liu et al., 2014).

Generally, analysis of the 16S rRNA sequencing data typically begins by grouping them into operational taxonomic units (OTUs) (Turnbaugh et al., 2007; Peterson et al., 2009; Větrovský et al., 2018) that contain similar 16S rRNA sequences with high sequence similarity (Seguritan and Rohwer, 2001; Enright et al., 2002; Yooseph et al., 2007; Niu et al., 2010; Westcott and Schloss, 2017). OTUs can represent the microbial taxa and facilitate the downstream analysis for the calculation and visualization of diversity and composition of the microbes (Niu et al., 2011; Zorita et al., 2015; Zou et al., 2018). Thus, picking OTUs has become the backbone in the established workflows, such as QIIME2 (Caporaso et al., 2010; Bolyen et al., 2019), mothur (Schloss et al., 2009), and RDP tools (Wang et al., 2007; Cole et al., 2009, 2013), which are used to analyze the microbial community structures.

In the last decade, a growing number of clustering methods have been proposed to cluster the 16S rRNA sequences into OTUs. However, different methods produce quite diverse outputs, even though a slight parameter change for the same method can also generate distinct results. A more general problem faced by microbial researchers is how to select one suitable method to obtain better clustering results. Therefore, understanding the principle and performance of different clustering algorithms is crucial for users to employ one suitable method for analyzing their sequence data. In this review, we summarized existing state-of-the-art clustering algorithms, explained their clustering mechanisms, analyzed their characters, compared their clustering performance on several benchmark datasets, and recommended some directions for developing new clustering algorithms. We hope this review can assist the biological researchers to select a reasonable clustering method for analyzing their collected sequences and help algorithm developers to design more efficient sequence clustering methods.

## Methods of Operational Taxonomic Unit Picking

Numerous OTU picking methods have been developed, which can be categorized as closed-reference clustering, *de novo* clustering (also called taxonomy independent), and open-reference clustering (Lawley and Tannock, 2017; Whelan and Surette, 2017; De Filippis et al., 2018). The closed-reference clustering involves comparing each query sequence to an annotated reference taxonomy database by utilizing the sequence classification or searching methods (Liu et al., 2017, 2018; Matias Rodrigues et al., 2017; Wei et al., 2020), then sequences matched to the same reference sequence are grouped into the same OTU. However, if a large portion of microbes in a sample has not yet been well defined, that is, not recorded in databases (i.e., unknown taxa), then they cannot be assigned to an OTU. Thus, closed-reference clustering methods are largely dependent on the completeness of the reference database, hence, have a poor performance on the condition that many novel organisms exist in the sequencing data (Schloss and Westcott, 2011; Chen et al., 2016). Furthermore, two query sequences matched to the same reference sequence may have a lower similarity to each other (Westcott and Schloss, 2015). As a result, closed-reference methods are often applied for the purpose of sequence annotation (Sun et al., 2011). For *de novo* clustering, all sequences are clustered into OTUs based on the pairwise sequence distances rather than comparing against a reference database (Forster et al., 2016). That is, *de novo* clustering methods compare each sequence against each other, followed by implementing different clustering algorithms at a specified threshold to group sequences into OTUs. For the open-reference clustering, it is a combination of the closed-reference and *de novo* methods. Here, a closed-reference clustering approach is first used to assign OTUs, and the unassigned sequences outputted by the closed-reference approach are then grouped by a *de novo* clustering method. Open-reference clustering blends the strengths and weaknesses of the other method and adds the complication that closed-reference and *de novo* clustering use different OTU definitions (Westcott and Schloss, 2017). As a result, *de novo* clustering does not depend on any reference database and, hence, can assign all sequences into OTUs, including both sequences that have been deposited in annotated databases as well as novel unknown ones (Zou et al., 2018). Additionally, several studies (Jackson et al., 2016; Schloss, 2016) also show that *de novo* clustering methods significantly outperform the other two approaches for picking OTUs. Therefore, *de novo* clustering attracts more attention and has become the preferred choice for researchers (Schloss, 2010; Cai et al., 2017). In the following, we mainly focus on *de novo* clustering.

Many different *de novo* clustering methods have been proposed to pick OTUs in the past decade, which can be further classified into four general categories: hierarchical clustering, heuristic clustering, model-based, and network-based clustering methods.

### Hierarchical Clustering Methods

**FIGURE 1:**
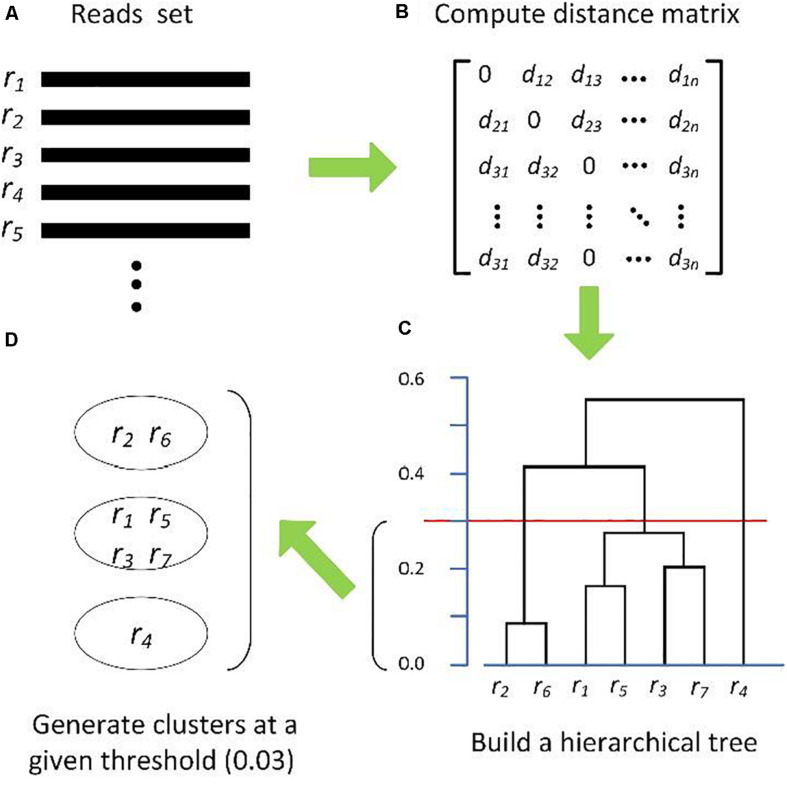
Schematic diagram of hierarchical clustering algorithms. **(A)** Input reads set, **(B)** distance matrix, **(C)** hierarchical Tree, and **(D)** OTUs formation.

**FIGURE 2:**
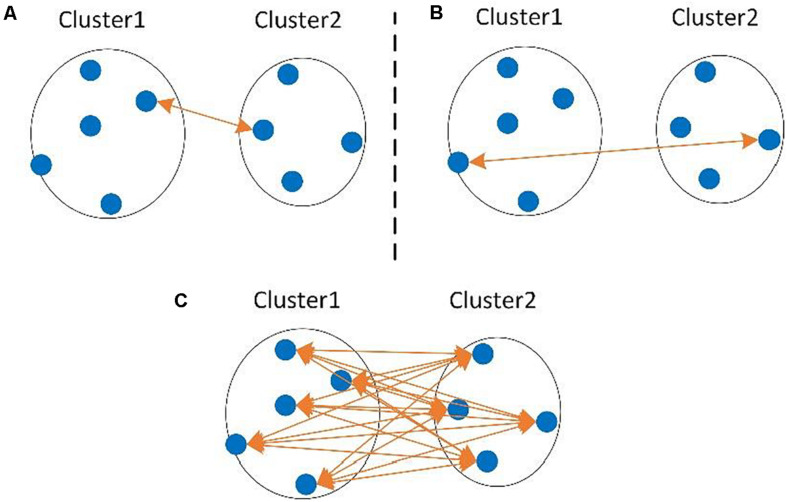
The distance between two clusters defined in single-linkage (SL) **(A)**, complete-linkage (CL) **(B)**, and average-linkage (AL) **(C)** clustering algorithms.

Hierarchical clustering methods generally require a full distance matrix between all sequences based on pairwise sequence alignment or multiple sequence alignment, then construct a hierarchical tree on the distance matrix. By applying a predefined clustering threshold to the hierarchical tree, sequences within the threshold are grouped into one OTU, as shown in Figure 1. Actually, most hierarchical methods implement the complete-linkage (CL), average-linkage (AL), or single-linkage (SL) algorithms (Zhang and Wei, 2015). CL, SL, and AL belong to the agglomerative methods, that is, in the beginning, each sequence is one cluster, then compute the similarity (i.e., distance) between each of the clusters and merge the two most similar clusters. Repeat the previous step until there is only a single cluster left, or the merging distance meets the given threshold (Figure 1C). The main differences among CL, SL, and AL are the distance criteria defined between two clusters (Figure 2), which can reflect the degree of clustering. For SL, the distance between two clusters is the minimum distance between two sequences in each cluster (Figure 2A). For CL, the distance between two clusters is defined as the maximum distance between two sequences in each cluster (Figure 2B). For AL, the distance between two clusters is defined as the average distance between each sequence in one cluster to every sequence in the other cluster (Figure 2C). We can see that SL is a loose clustering strategy, CL is the most stringent, and AL is the middle ground between SL and CL.

DOTUR (Schloss and Handelsman, 2005) is probably the first published tool for hierarchically clustering sequences into OTUs by using CL, AL, and SL. mothur (Schloss et al., 2009), the improved version of DOTUR, has become the representative hierarchical clustering method for picking OTUs. As with DOTUR, mothur needs to load the distance matrix into computer memory before performing clustering. In order to alleviate the computational complexity and memory usage, Sun et al. (2009) proposed a novel algorithm (namely, ESPRIT), which adopts the *k*-mer (substrings of length *k*) distance to rapidly identify the sequence pairs with high similarity and stores the reduced distance by using a sparse matrix. In the procedure of picking OTUs, the Hcluster algorithm was devised to perform CL clustering, which can avoid loading the whole matrix into memory. Huse et al. (2010) observed that the CL algorithm is sensitive to sequencing artifacts, then they proposed a single-linkage preclustering (SLP) to overcome the effect of sequencing errors and decrease the inflation of OTUs. Cole et al. (2013) proposed the mcClust algorithm to achieve the CL strategy that allows the distance matrix computation to be parallelized, which can lower the time complexity. Matias Rodrigues and von Mering (2013) presented the HPC-CLUST pipeline, a distributed implementation of two hierarchical clustering algorithms (CL and AL) with high optimization. HPC-CLUST takes as input a set of pre-aligned sequences and efficiently allocates both memory usage and computing complexity, which can handle large numbers of sequences on a computer cluster. Franzén et al. (2015) developed the oclust method in which the distance matrix and CL clustering are performed with an R package based on the pre-aligned sequences. Similar to the HPC-CLUST, the oclust also needs to pre-align sequences, which is usually computation intensive.

Generally, the computational complexity of hierarchical algorithms both in time and space is *O*(*N*^2^), where *N* is the number of sequences. Thus, the computational cost of most hierarchical methods quadratically scales with the number of sequence increases. As a result, hierarchical clustering methods are not suitable for handling huge numbers of sequences because of their intrinsic computing complexity (Barriuso et al., 2011).

### Heuristic Clustering Methods

**FIGURE 3:**
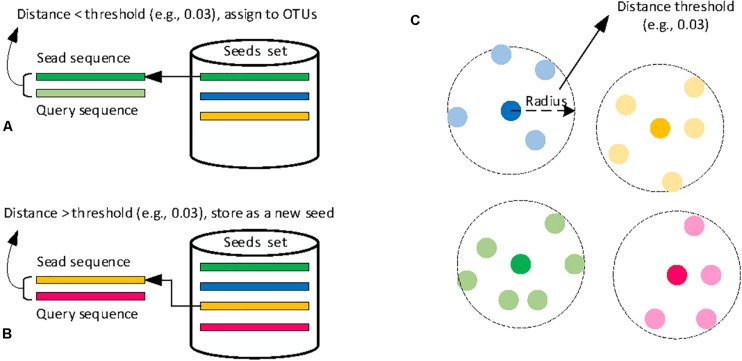
Schematic diagram of classical heuristic clustering methods. **(A)** sequence assignment, **(B)** new seed generation, and **(C)** OTUs results.

Heuristic clustering processes input sequences one by one, avoiding the expensive step of computing distances of all pairwise sequences. Most classical heuristic clustering methods use pairwise sequence alignment and generate clusters in a greedy incremental strategy (GIS), which is shown in Figure 3. These methods use one sequence (called seed) to represent its cluster, and each query sequence is compared with all seeds of existing clusters (Chen et al., 2016). One query sequence is assigned to a cluster if the distance between the sequence and one seed meets the clustering threshold (Figure 3A). Otherwise, a new cluster is created, and the query sequence becomes the seed sequence (Figure 3B). Due to the comparison of all sequences just with the seeds of clusters, greedy heuristic clustering is computationally much more efficient than hierarchical clustering methods. As a result, many different heuristic clustering algorithms have been developed, and the main differences are the seed selection and distance calculation.

CD-HIT (Li and Godzik, 2006; Huang et al., 2010; Fu et al., 2012) and USEARCH (Edgar, 2010) are the two best-known heuristic methods for picking OTUs. The main discrepancy between these two methods is the sequence sorting before clustering. CD-HIT sorts by the length of sequences while USEARCH by sequence abundance. UPARSE (Edgar, 2013) is an improved version of USEARCH, which adds the chimera detection for seed sequences. Different from sequence distance calculation in CD-HIT and USEARCH, GramClust (Russell et al., 2010) designs a distance metric based on the inherent grammar of each pairwise sequences for clustering a set of sequences. DNACLUST (Ghodsi et al., 2011) also follows the GIS way, but it uses a novel *k*-mer-based filtering algorithm to accelerate the clustering procedure. Similar to DNACLUST, LST-HIT (Namiki et al., 2013) introduces a new filtering scheme to remove dissimilar sequence pairs on the basis of the longest common subsequence before performing pairwise sequence alignment, which can speed up the computation. SUMACLUST (Mercier et al., 2013) and OTUCLUST (Albanese et al., 2015) are another two greedy clustering methods that are designed to perform exact sequence alignment, rather than semiglobal alignments implemented in CD-HIT and USEARCH. Additionally, OTUCLUST performs sequence de-duplication and chimera removal. LSH (Rasheed et al., 2013) is also another greedy clustering algorithm that utilizes the locality-sensitive hashing to accelerate the pairwise sequence comparisons and incorporates a matching criterion to improve the quality of sequence comparisons. Considering that using a single global clustering threshold is too relaxed for slow-evolving lineages, Mahé et al. (2014) designed Swarm, which first generates an initial set of OTUs by iteratively agglomerating similar sequences, then breaks them into sub-OTUs to refine the clustering results by using abundance information and OTUs’ internal structures. VSEARSH (Rognes et al., 2016) is a free 64-bit and open-source versatile program and is designed as an alternative to the USEARCH tool for which the source code is not publicly available and only a memory-confined 32-bit version is freely available for academic users.

The above heuristic methods just select one sequence as the seed to represent the cluster. Once the seed is selected, it will not be changed anymore, resulting in the outcomes sensitive to the selected seeds. Therefore, how to select a “good” seed that includes more cluster information is significantly important. Some methods have been proposed to achieve this target. Zheng et al. (2012) introduced a dynamic seed-based clustering method (namely, DySC) to reselect seed sequences. DySC first uses the traditional GIS to form the pending clusters. Once a pending cluster reaches a threshold size, it is converted into a fixed cluster, and a new fixed seed is reselected, which is defined as the sequence that maximizes the sum of *k*-mers shared between the fixed read and other reads in one cluster. Chen et al. (2013a) proposed MSClust, a multiseed-based heuristic clustering method. The multiseeds for one cluster are generated based on an adaptive strategy, that is, one query sequence is assigned to one cluster if the average distance between the sequence and seeds is smaller than the user-defined threshold; otherwise, the sequence is marked as unassigned. In order to reduce the sensitivity of seeds to sequencing errors, we developed DBH (Wei and Zhang, 2017), a de Bruijn (DB) graph-based heuristic clustering method. It first forms temporary clusters using the traditional GIS. When the size of a temporary cluster reaches the predefined minimum sequence number, DBH builds a DB graph for this cluster and generates a new seed to represent this cluster. Finally, the remaining sequences are assigned to the corresponding OTUs. Later, We designed DMSC (Wei and Zhang, 2019), a dynamic multiseed clustering method for OTU picking. DMSC first generates a series of clusters based on the GIS strategy. When the sequence number in a cluster is larger than the value of a predefined size, the multicore sequence (MCS) selection procedure is triggered, and the MCS is applied as the seeds of the cluster. The MCS is determined as the *n*-core sequences (*n* ≥ 3) that the distance between any two sequences in the MCS is less than the clustering threshold. If a new sequence is added to one cluster according to the average distance to MCS and the distance standard deviation in MCS, DMSC will update the MCS. By reselecting seed sequences, these four methods can achieve higher clustering accuracy than the traditional heuristic methods such as CD-HIT and USEARCH. Recently, Bazin et al. (2018) proposed a fuzzy OTU-picking algorithm that adds the uncertainty information to the clustering based on fuzzy sets, which can also improve the clustering quality.

Different from most existing clustering methods that use the seed sequences to represent clusters, Cai and Sun (2011) developed the ESPRIT-Tree method, which initially constructs a PBP (pseudometric-based partition) tree that provides a coarse representation of the entire sequences, then iteratively finds the closest pairs of sequences or clusters and merges them into a new cluster. Later, they proposed an improved method of ESPRIT-Forest (Cai et al., 2017), which can cluster massive sequence data in a subquadratic computational complexity. Pagni et al. (2013) introduced DBC454 for clustering ITS1 (fungal internal transcribed spacer 1) sequences using a density-based hierarchical clustering procedure. Recently, Westcott and Schloss (2017) designed OptiClust that maximizes the value of Matthews correlation coefficient (MCC) by iteratively reassigning sequences to new OTUs.

Broadly speaking, heuristic clustering methods have a lower computational complexity of *O*(*KN*), where *K* is the final number of clusters. Usually *K* ≤ *N*, and hence, heuristic clustering methods are computationally much more efficient than hierarchical clustering methods and are more widely employed to deal with hundreds of thousands of 16S rRNA sequences.

### Model-Based Clustering Methods

One of the critical problems with most existing hierarchical and heuristic clustering methods is the need to select a constant and optimal distance threshold to define OTUs at a distinct taxonomic level (e.g., species). A slight change in threshold can result in very different OTUs. Model-based clustering methods, such as CROP (Hao et al., 2011), BEBaC (Cheng et al., 2012), and BC (Jääskinen et al., 2014), were developed to address this issue. CROP (Hao et al., 2011) builds a Bayesian model to cluster sequences, which utilizes a Gaussian mixture model and a birth–death process to characterize a specific cluster. BEBaC (Cheng et al., 2012) first uses the heuristic trick to assign the highly similar sequences to form a pregroup, then similar 3-mer count vectors are assigned into crude clusters by searching for the best partitions that achieve the maximum posterior possibility for given sequence data. In the fine clustering phase, BEBaC applies a minimum description length criterion to determine the number of OTUs, generating the final partitioning. BC (Jääskinen et al., 2014) first models the sequences using Markov chains, then uses a Bayesian partition model with the Dirichlet process to split and merge clusters. Although these methods partition sequences into OTUs without additional information besides the sequence data itself, it is not suitable for large-scale sequence datasets.

### Network-Based Clustering Methods

**FIGURE 4:**
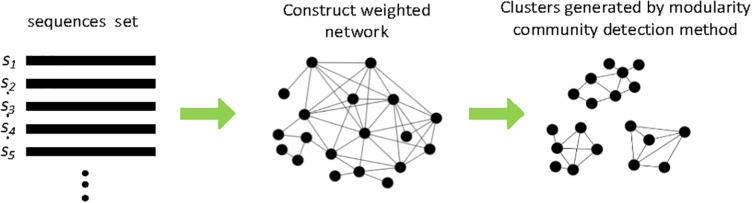
Schematic diagram of network-based methods.

Several network-based clustering methods such as M-pick (Wang et al., 2013), MtHc (Wei and Zhang, 2015), and DMclust (Wei et al., 2017) were also proposed to solve the problem of requiring a given clustering distance to pick OTUs. Figure 4 shows the schematic diagram of the main processing steps in network-based clustering methods. M-pick (Wang et al., 2013) first compute the distances across all pairs of sequences to construct a fully connected graph, then prunes the complete graph to generate a neighborhood graph; finally, a modularity-based community detection approach is recursively performed to form OTUs. Based on the concept of network motif, we proposed MtHc (Wei and Zhang, 2015). MtHc first searches for sequence motifs using a heuristic strategy then uses these sequence motifs as seeds to generate candidate clusters, which are hierarchically merged into OTUs based on the distances of motifs between two clusters. Later, we developed DMclust (Wei et al., 2017); it first searches for the sequence dense groups, which are viewed as nods to construct a weighted graph, then a modularity-based clustering method is applied to capture the community structures in sequence data to generate clusters.

Network-based methods require a full distance matrix of all pairwise sequences to construct a graph and, hence, has a high computational complexity in terms of run time and memory usage. They cannot handle large numbers of sequences.

**FIGURE 5:**
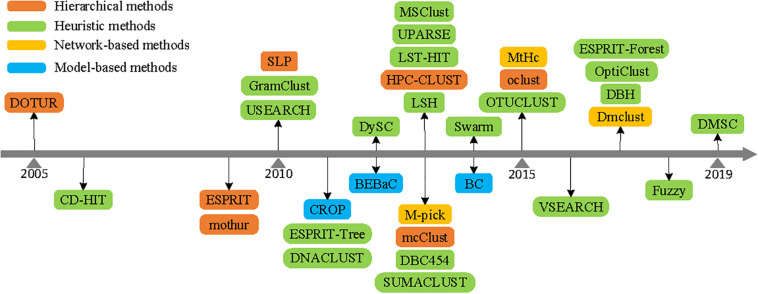
Published years of operational taxonomic unit (OTU) picking methods (mentioned in this paper).

Based on the above analysis, Figure 5 describes the development history of clustering methods according to their published years. It can be summarized that hierarchical clustering (either based on AL, SL, or CL) and network-based clustering methods need to compute and store a full distance matrix of all pairwise sequences, adding the computational complexity and memory space usage. Although the model-based clustering method could produce better clustering results, their run time would render them unusable on massive quantities of sequences. Due to the comparison of each sequence just with the seed sequences, heuristic clustering methods are capable of handling millions of sequences and are more widely employed to analyze massive 16S rRNA datasets (Cai and Sun, 2011). With the sequencing technology development, the volume of sequences increases drastically, and heuristic clustering methods continue to attract more attention in picking OTUs.

## Materials of Benchmark Datasets and Evaluation Metrics

### Benchmark Datasets

Three benchmark studies, including one simulated and two real-world sequence datasets, were conducted to assess the performance of 12 existing OTU-picking algorithms. The simulated dataset was directly produced by Seq-Gen (Rambaut and Grass, 1997) sequence simulator. It can be directly downloaded from BEBaC (Cheng et al., 2012). Two real-life sequence datasets are the V4 hypervariable region dataset from the murine gut and the global 16S bacterial rRNA gene sequence dataset, respectively. These sequence datasets have been widely used to validate the performance of clustering results (Cheng et al., 2012; Wei and Zhang, 2017, 2019).

For the simulated dataset, the ground truths (labels of sequences) are directly taken from simulated data, in which we exactly know the species of each sequence. However, for real-life datasets, we need to construct the ground-truth information by searching a reference database. The processing procedures of obtaining ground truth information for real-life datasets are described in Supplementary Figure S1. First, the V4 pair-end sequencing data are merged by the FLASH (Magoè and Salzberg, 2011) assembly tool. Then, the merged sequences are cleaned to remove sequences with low quality and short length by quality USEARCH (Edgar, 2010) filtering software. The Python executive command (*assign_taxonomy.py*) in QIIME (Caporaso et al., 2010) is applied to align the cleaned sequences to the default reference database (Greengenes DeSantis et al., 2006) to obtain the species information. Last, aligned sequences with high alignment quality (i.e., >97% identity over an aligned region >90% of the length of the sequences) are retained, and the remaining annotated sequences are adopted to construct the final ground-truth. These procedures of constructing the ground-truth information are based on previous studies (Cai and Sun, 2011; Wei and Zhang, 2019). Some detailed features (such as taxon number, sequences number, and average sequence length) of three benchmark datasets are listed in the following Table 1.

**TABLE 1:** Statistics of three benchmark datasets for operational taxonomic unit (OTU) picking.

**Sequence data**	**Taxon number**	**Total sequences**	**Average length**	**Variable regions**	**References**
Simulated dataset	9	22 K	500	-	Cheng et al., 2012
V4 dataset	68	∼511 K	253	V4	Westcott and Schloss, 2015
Global 16S rRNA	1,498	∼887 K	∼1,400	V1-V9	Matias Rodrigues and von Mering, 2013

### Evaluation Metrics

The number of OTUs, normalized mutual information (NMI), Matthews correlation coefficient (MCC), adjusted rand index (ARI), and adjusted mutual information (AMI) metrics are used to evaluate the clustering performance. OTU number is the cluster number that directly inflects the count of species (or genera). NMI value is commonly applied to estimate the clustering accuracy, that is, how the outcome of one clustering algorithm agrees with the ground truth (Chen et al., 2013b). ARI (Nguyen et al., 2015; Jin and Bi, 2018) represents the number of pairwise sequences that are either in the same cluster or in different clusters in both partitions. AMI is similar to ARI. Different from NMI, AMI, and ARI that rely on an external reference, the metric of MCC can be calculated according to the clustering threshold and distances between sequences (Schloss and Westcott, 2011); thus, MCC is regarded as an objective criterion to evaluate the clustering quality of different algorithms for OTU picking (Westcott and Schloss, 2015; Schloss, 2016; Liu et al., 2019). AMI, ARI, and MCC vary between -1 and 1, and a larger value represents better clustering quality. How to calculate these metrics are provided in the Supplementary File.

## Comparison Results

We evaluate 12 state-of-the-art OTU picking methods, that is, CD-HIT (v.4.6.8) (Li and Godzik, 2006), USEARCH (v.11.0.667) (Edgar, 2010), DNACLUST (Ghodsi et al., 2011), Swarm (v.1.2.19) (Mahé et al., 2014), VSEARCH (v.2.3.4) (Rognes et al., 2016), DBH (Wei and Zhang, 2017), DMSC (Wei and Zhang, 2019), DySC (v.06-1-2012) (Zheng et al., 2012), ESPRIT-Forest (Cai et al., 2017), GramClust (v.1.3) (Russell et al., 2010), average linkage (AL) clustering method employed in mothur software (v.1.44.3) (Schloss et al., 2009), and CROP (Hao et al., 2011). Among these methods, CD-HIT, USEARCH, DNACLUST, Swarm, VSEARCH, DySC, ESPRIT-Forest, DBH, GramClust, and DMSC are the typical heuristic clustering methods; mothur is a comprehensive software package for sequence clustering, and it is demonstrated that the AL clustering implemented in mothur (mothur-AL) is a reliable method to represent the actual distances between sequences (Westcott and Schloss, 2015); CROP is a model-based method. All methods were executed on the same Linux server for OTU picking. The running parameters and command lines of each algorithm are given in Supplementary Table S1.

### Benchmarking on the Simulated Dataset

**FIGURE 6:**
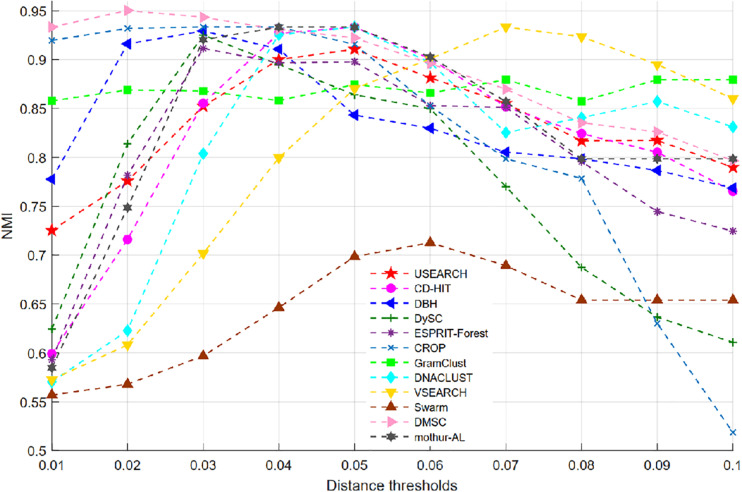
Normalized mutual information (NMI) values of different clustering methods on the simulated dataset.

Figure 6 shows the NMI values of 12 clustering methods as a function of distance thresholds ranging from 0.01 to 0.1. Because Swarm does not apply the distance threshold to cluster, and just uses the parameter *d* (*d* nucleotide differences) to generate OTUs, the setting of *d* is calculated by *d* = *d*_th_ × *L*_ave_, where *L*_ave_ is the average length (i.e., 500) of this simulated data, *d*_th_ is the distance threshold ranging from 0.01 to 0.1. From Figure 6, we can see that all methods, except VSEARCH and GramClust, show a similar trend, that is, they achieved higher NMI values near 0.04 distance but lower NMI when the distance threshold increases. The NMI peak values of the different methods occur at different distance thresholds. This is mainly due to the discrepancies of distance calculation and clustering strategy in each method. VSEARCH shows a different trend from other methods. It obtained the NMI peak at 0.07 distance, while the other methods achieved their NMI peak value near 0.04 distance. The NMI values of GramClust is always between 0.85 and 0.90 even in lower distances. The peak NMI scores of 11 methods and the corresponding inferred OTU number at different distance thresholds are reported in Table 2. It can be found that DMSC, CROP, DBH, CD-HIT, VSEARCH, DNACLUST, Swarm, GramClust, and mothur-AL successfully generated nine OTUs at their maximum NMI values, while USEARCH, DySC, and ESPRIT-Forest overestimated OTUs.

**TABLE 2:** Maximum normalized mutual information (NMI) values for different OTU picking methods on the simulated dataset.

	**DMSC (0.02)**	**USEARCH (0.05)**	**DySC (0.03)**	**ESPRIT-Forest (0.05)**	**CD-HIT (0.05)**	**CROP (0.03)**
Max. NMI	0.9503	0.9107	0.9252	0.8979	0.9334	0.9334
OTUs number	9	10	17	13	9	9

	**VSEARCH (0.07)**	**DNACLUST (0.05)**	**Swarm (*d* = 15)**	**GramClust (0.07)**	**DBH (0.03)**	**Mothur-AL (0.04)**

Max. NMI	0.9334	0.9333	0.9334	0.8795	0.9293	0.9333
OTUs number	9	9	9	9	9	9

**The value in the parentheses is the clustering threshold where each method achieves its peak NMI. For the Swarm method, it is the value of parameter d.**

**FIGURE 7:**
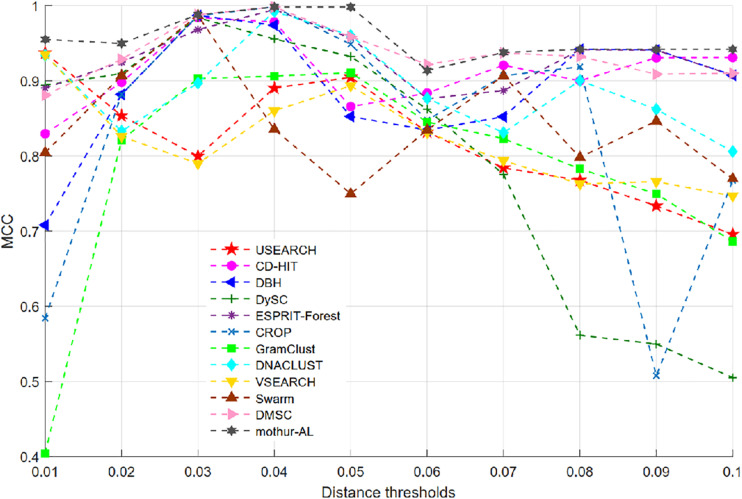
The Matthews correlation coefficient (MCC) values of 12 OTU picking methods on the simulated dataset.

Figure 7 illustrates the MCC values of 12 OTU picking methods at different clustering thresholds. Similar to the NMI curve, all methods achieved the highest MCC value near 0.04 distance threshold, while USEARCH and VSEARCH obtained their MCC peak values at 0.01 distance. Table 3 reports the average, standard deviation (SD), and maximum of MCC scores with the inferred OTUs number. It can be observed that DMSC, CROP, Swarm, GramClust, DBH, and mothur-AL methods also can produce the exact OTU number at their best MCC values, while USEARCH, DySC, ESPRIT-Forest, CD-HIT, VSEARCH, and DNACLUST overestimated the OTU number. Based on the MCC values listed in Table 3, we can see that DMSC, ESPRIT-Forest, CD-HIT, and mothur-AL have a better clustering quality (ave. MCC > 0.9) than other methods, and mothur-AL has the best average MCC value. The NMI values, OTUs number, and MCC values of the different methods in the range of 0.01–0.1 distance thresholds can be seen in Supplementary Table S2.

**TABLE 3:** The average, SD, and maximum MCC values of 11 OTU picking methods on the simulated dataset.

	**DMSC (0.04)**	**USEARCH (0.01)**	**DySC (0.03)**	**ESPRIT-Forest (0.04)**	**CD-HIT (0.03)**	**CROP (0.03)**
Max. MCC	0.9980	0.9369	0.9838	0.9947	0.9840	0.9980
OTUs number	9	528	17	16	27	9
Ave. MCC	0.9363	0.8198	0.7929	0.9286	0.9120	0.8347
SD of MCC	0.0343	0.0737	0.1750	0.0366	0.0451	0.1585

	**VSEARCH (0.01)**	**DNACLUST (0.04)**	**Swarm (*d* = 15)**	**GramClust (0.05)**	**DBH (0.03)**	**Mothur-AL (0.04)**

Max. MCC	0.9349	0.9921	0.9868	0.9106	0.9868	0.9980
OTUs number	1,291	15	9	9	9	9
Ave. MCC	0.8204	0.8891	0.5474	0.7832	0.8879	0.9564
SD of MCC	0.0578	0.0567	0.1385	0.1436	0.0781	0.0270

**The value in the parentheses is the clustering threshold where each method achieves its peak MCC. For the Swarm method, it is the value of parameter d.**

Supplementary Figures S2, S3 depict the ARI and AMI curves of 12 OTU picking methods at different clustering thresholds. On the whole, the curves of ARI and AMI are similar to those of NMI. That is, most methods, e.g., CD-HIT, DBH, DySC ESPRIT-Forest, DNACLUST, Swarm, DMSC, and mothur-AL obtained higher ARI and AMI values near 0.04 distance but lower ARI when the distance threshold increases, while VSEARCH and UCLUST show a different trend from other methods where they obtained the ARI peak at 0.07 distance. The ARI values of GramClust are always between 0.65 and 0.67 even in lower distances, and AMI values are between 0.79 and 0.81. Although CROP achieved the highest ARI (at 0.01 distance threshold) among all methods, it generated 158 OTUs, 17 times larger than the true number. The maximum ARI and AMI values of the 11 methods at different clustering thresholds are listed in Supplementary Tables S3, S4. It can be found that some clustering methods (such as DMSC, VSEARCH, DNACLUST, Swarm, GramClust, DBH, and mothur-AL) can exactly infer the true number of OTUs at their best ARI and AMI values for the simulated dataset.

### Benchmarking on V4 Dataset

**FIGURE 8:**
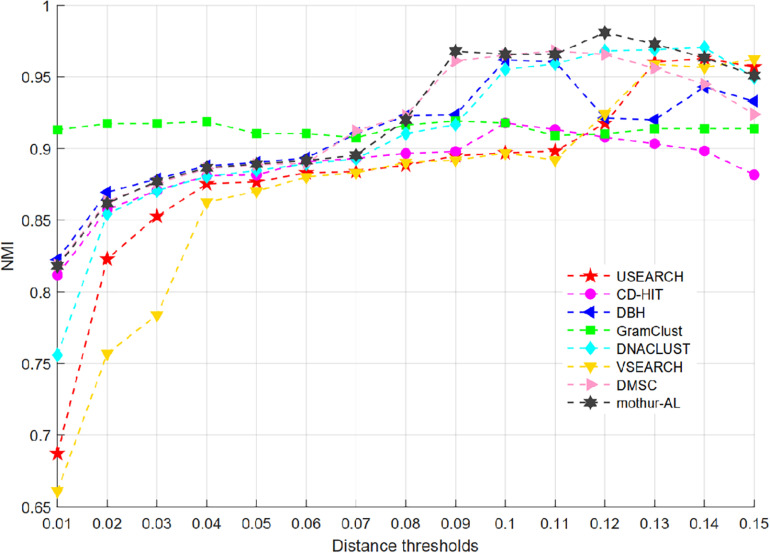
NMI values of eight OTU picking methods at different clustering thresholds on the V4 dataset.

For the V4 dataset, just eight methods of USEARCH, CD-HIT, DBH, GramClust, DNACLUST, VSEARCH, DMSC, and mothur-AL can generate the clustering results at each distance threshold, while ESPRIST-Forest, DySC, CROP, and Swarm cannot handle this dataset. Figure 8 shows the NMI curves of each clustering method, and Supplementary Figure S4 presents the inferred OTU number at different clustering thresholds. We can see that GramClust has higher NMI scores than other approaches when the distance increases from 0.01 to 0.06. DMSC and mothur-AL have higher NMI values than the other methods at distance thresholds from 0.09 and 0.11, and mothur-AL achieved the highest NMI score at 0.12 threshold. For the OTU number in Supplementary Figure S4, all methods show a similar descending trend from 0.01 to 0.15, generating close OTU number to the ground truth near 0.1 distance except GramClust and mothur-AL. mothur-AL obtained close OTU number at 0.08 distance threshold. GramClust produces more OTUs than the ground truth even in low distance thresholds. The ARI and AMI curves of each clustering method are described in Supplementary Figures S5, S6, which show a similar result to the curve of NMI.

**FIGURE 9:**
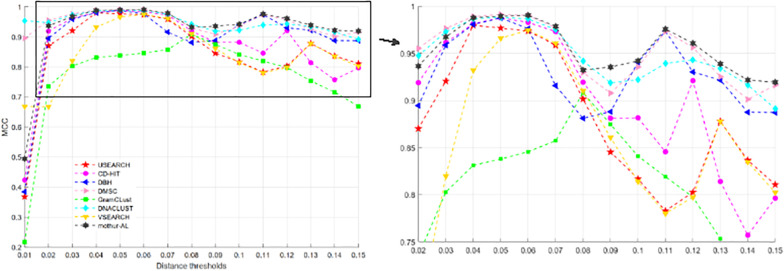
MCC values of eight OTU picking methods with different clustering thresholds on the V4 dataset.

Figure 9 describes the MCC values at different distance thresholds, and Table 4 reports the maximum, average, and SD of MCC values for each method. Obviously, from Table 4, we can find that DMSC, DNACLUST, and mothur-AL achieve higher average MCC values than other clustering methods, indicating that these three methods can produce higher clustering quality on the V4 dataset. The NMI values, OTU number, MCC, ARI, and AMI values of each method with different distance thresholds can be found in Supplementary Tables S5, S6.

**TABLE 4:** The average, SD, and maximum MCC values of seven OTU picking methods on V4 dataset.

	**DMSC (0.05)**	**USEARCH (0.04)**	**VSEARCH (0.06)**	**DNACLUST (0.05)**	**DBH (0.05)**	**GramClust (0.08)**	**CD-HIT (0.05)**	**mothur-AL (0.06)**
Max.	0.9913	0.9797	0.9746	0.9884	0.9875	0.9083	0.9876	0.9904
Ave.	0.9480	0.8481	0.8444	0.9478	0.8938	0.7671	0.8697	0.9246
SD	0.0330	0.1438	0.0933	0.0283	0.1409	0.1593	0.1382	0.1175

**The values in the parentheses are the clustering thresholds where each method achieves its peak MCC.**

### Benchmarking on Global 16S rRNA Sequence Dataset

The global 16S rRNA dataset was often employed to test the scalability of dealing with longer sequences. For this near full-length 16S dataset, only USEARCH, CD-HIT, VSEARCH, and DBH can get the clustering results. Other methods fail to hand with this large-scale dataset.

The NMI values of USEARCH, CD-HIT, VSEARCH, and DBH with different clustering thresholds are shown in Supplementary Figure S7. We can observe that CD-HIT achieves higher NMI scores than other approaches at distance thresholds from 0.01 to 0.07, while USEARCH and VSEARCH obtain higher NMI scores than DBH and CD-HIT with distance increases from 0.11 to 0.15. The AMI values of USEARCH, CD-HIT, VSEARCH, and DBH are described in Supplementary Figure S8, which shows a similar result to the NMI values in Supplementary Figure S7. Supplementary Figure S9 represents the OTU number inferred by these four methods. It can be seen that four OTU picking methods present a similar trend, that is, the OTU number exponentially decreases when the clustering distance increases. Four OTU picking methods of USEARCH, CD-HIT, VSEARCH, and DBH overestimate OTUs in the distance range from 0.01 to 0.13. Supplementary Figure S10 shows the ARI values of USEARCH, CD-HIT, VSEARCH, and DBH. We can see that CD-HIT achieves higher ARI values than other methods at distance thresholds from 0.01 to 0.07, DBH obtains the highest ARI at distance thresholds from 0.08 to 0.10, while USEARCH and VSEARCH obtain higher NMI scores than DBH and CD-HIT with distance ranging from 0.12 to 0.15. Supplementary Figure S11 describes the MCC values of four OTU picking methods. Obviously, DBH achieves higher MCC values than CD-HIT, USEARCH, and VSEARCH at any distance threshold, indicating that DBH can produce better clustering quality for this full-length 16S rRNA dataset. The NMI, MCC, ARI, AMI values, and OTU number of each method are provided in Supplementary Table S7.

### Computational Complexity Analysis

Finally, in order to evaluate the computational complexity (including running time and memory usage) of different OTU picking methods, we used one large volume sequence dataset (V35) processed by QIIME from the HMP official website^2^, which covers V3–V5 hypervariable regions and contains ∼30.3 million sequences with an average length of 528 bp. It is reported that with sequencing coverage or sequences increase, the probability of duplicate sequences will be observed (Schloss and Westcott, 2011). Thus, for relatively fair comparisons across different OTU picking algorithms, the unique sequences (∼19.8 million) of V35 were used to evaluate the computational complexity of the OTU picking methods. We only report the computational complexity of nine heuristic methods of CD-HIT, DBH, DMSC, DNACLUST, DySC, GramClust, Swarm, USEARCH, and VESARCH because mothur-AL and CROP are time consuming for large-scale datasets, and ESPRIT-Forest always returns a core dumped information. Supplementary Figure S12A depicts the execution time (wall time) of nine OTU picking algorithms with different sequence sizes ranging from 10^4^ to 10^6^. It can be seen that the speed of DMSC is lower than that of other clustering methods. The speed of DBH, USEARCH, DNACLUST, and CD-HIT is faster than other methods when the sequence number increases. Supplementary Figure S12B graphically describes the memory usage for each method. We can obverse that DMSC and VESARCH consume more memory than other clustering methods, while Swarm, DySC, GramClust, and CD-HIT need less memory usage than other methods.

## Conclusion and Perspectives

With the development of high-throughput sequencing technologies, it has become convenient and cost efficient to thoroughly profile the microbial community composition and diversity in various environmental habitats (Deshpande et al., 2018; Escalona et al., 2018; Rodriguez-R et al., 2018; Fritz et al., 2019; Huang et al., 2021). Millions of sequencing data can be generated, and how to utilize this enormous sequence resource has become a critical concern for microbial ecologists (Szalkai and Grolmusz, 2018; Qu et al., 2019b). One particular challenge is the OTU picking in amplicon sequence analysis. Luckily, this challenge can be directly addressed by sequence clustering that attempts to group similar sequences (De Vrieze et al., 2018; Edgar, 2018). Therefore, numerous clustering methods have been proposed to help to unlock the great wealth contained in sequence datasets, but none of the methods notably outperforms all the others, and how to choose an appropriate method has become a challenge for inexperienced users. A lot of time and resources can be wasted in selecting clustering tools and analyzing the clustering results. In this review, we introduced the recent advance of clustering methods, which mainly focuses on three aspects: (i) the principles of existing clustering algorithms, (ii) benchmark dataset construction for OTU picking and evaluation metrics, and (iii) the performance of different methods with various similarity/distance thresholds on benchmark datasets. From the scope of clustering algorithms, we introduced the key clustering procedures for each category, such as hierarchical clustering methods, heuristic clustering methods, model-based methods, and network-based methods. From the scope of benchmark dataset construction and evaluation metrics, we introduced how to construct the ground-truth information for real-life 16S rRNA sequence datasets, presenting different criteria to evaluate clustering methods.

We compared the performance of the existing 12 state-of-art OTU picking methods of CD-HIT, USEARCH, DNACLUST, Swarm, VSEARCH, DBH, DMSC, DySC, ESPRIT-Forest, GramClust, mothur-AL, and CROP. It is found that the performance of most methods with different distance thresholds shows similar clustering results in terms of NMI. DMSC, DNACLUST, and USEARCH achieved the NMI peak values on the simulated dataset, V4 dataset, and full-length 16S rRNA dataset, respectively. In terms of MCC, mothur-AL achieved better clustering results on simulated dataset, DMSC had better clustering results for V4 datasets, and DBH obtained better clustering results on the full-length 16S rRNA dataset. Although numerous OTU picking methods have been proposed, mothur still is a competitive tool for amplicon sequence analysis. Concomitant with the large number of sequences produced by high-throughput technologies, four future directions to design the OTU picking algorithms should be paid attention to. One direction is to design the powerful clustering methods for huge sequences with longer sequence length. A striking challenge brought by the advent of sequencing technology is the rapid growth of sequence length. Several third-generation sequencing technologies (e.g., PacBio, Nanopore) (Rhoads and Au, 2015; Han et al., 2018; Ono et al., 2020) claim to have a long read length of 10∼100 kbp, which can cover the whole region of 16S rRNA gen (Wagner et al., 2016; Pootakham et al., 2017; Earl et al., 2018). Therefore, OTU picking methods for longer sequences will be in high demand. Another is clustering stability. From the comparison results in terms of MCC, we can see that the MCC curve of each method varies a lot with the distance threshold changes. The MCC curve should be a straight line for a stable clustering method, that is, given different distance thresholds, the OTU picking method should cluster sequences within the distance threshold into one group and the sequences beyond the distance threshold into different groups. The third is the integration of new clustering algorithms to the popular sequence analysis platforms or pipelines, such as mothur and QIIME2. When an excellent clustering algorithm was developed, developers should let their algorithm be expandable or easy to be applied into the platforms, so that the clustering results or outputs of a new method can be directly used as the input of relative commands in platforms, or the outputs from the platforms can be directly fed into the new method. This will be very convenient for users to adopt new clustering algorithms in the platform. The last direction is how to handle sequencing errors (Ma et al., 2019). Most existing OTU picking methods are just designed for sequence clustering, while the sequences generated by the sequencing platform will inevitably contain sequencing errors (Gaspar, 2018). Removing or reducing the sequencing errors will improve the accuracy of describing the microbial community. Although some error-correction (denoising) methods, such as DATA2 (Callahan et al., 2016), UNOISE (Edgar, 2016), Deblur (Amir et al., 2017), and SeekDeep (Hathaway et al., 2017), have been developed, how to combine these error-correction methods with OTU picking methods needs attention.

## Data Availability Statement

The original contributions presented in the study are included in the article/Supplementary Material, further inquiries can be directed to the corresponding author/s.

## Author Contributions

Z-GW performed all the procedures using the clustering software, analyzed the clustering results, and wrote the manuscript. X-DZ downloaded the source codes and installed the software of all the clustering methods. MC and FL participated in the experimental studies and collected the benchmark datasets. YQ helped in improving the manuscript. S-WZ conceived the overall study, and reviewed and revised the manuscript. All authors read, edited, and approved the final manuscript.

## Conflict of Interest

The authors declare that the research was conducted in the absence of any commercial or financial relationships that could be construed as a potential conflict of interest.

## Footnotes

1 http://icomm.mbl.edu

2 https://www.hmpdacc.org/hmp/HMQCP/

## Abbreviations

AMI: adjusted mutual information
ARI: adjusted rand index (ARI)
AL: average linkage
CL: complete linkage
GIS: greedy incremental strategy
MCC: Matthews correlation coefficient
OTUs: operational taxonomic units
rRNA: ribosomal RNA
SL: single linkage
SD: standard deviation.
