# Supplementary material for: Comparison of Methods for Picking the Operational Taxonomic Units From Amplicon Sequences
Source: Front Microbiol. 2021 Mar 24;12:644012. doi: 10.3389/fmicb.2021.644012 (PMC8024490; doi:10.3389/fmicb.2021.644012)
Supplement: Supplementary file 1 [file Data_Sheet_1.docx]

**A comparison of methods for picking the operational taxonomic units from amplicon sequences**

Ze-Gang Wei^1,2^, Xiao-Dan Zhang^1^, Ming Cao^3,4^, Fei Liu^1^, Yu Qian^1^, Shao-Wu Zhang^2^*

1 Institute of Physics and Optoelectronics Technology, Baoji University of Arts and Sciences, Baoji, Shaanxi Province 721016, China

2 Key Laboratory of Information Fusion Technology of Ministry of Education, School of Automation, Northwestern Polytechnical University, Xi’an, Shaanxi Province 710072, China

3 Faculty of Electronic and Information Engineering, Xi'an Jiaotong University, Xi'an, Shaanxi Province 710049, China

4 School of Mathematics and Statistics, Shaanxi Xueqian Normal University, Xi'an, Shaanxi Province 710100, China

* To whom correspondence should be addressed. Email: zhangsw@nwpu.edu.cn

**Evaluation metrics**

**NMI calculation**

Suppose that there are *N* read sequences from *M* species $\Omega\text{=}\left\{t_{1},t_{2},...,t_{M}\right\}$and they are grouped into *C* OTUs $\Pi\text{=}\left\{o_{1},o_{2},...,o_{C}\right\}$ at a specific distance threshold with a given clustering algorithm. The NMI is calculated by the following equations [80].

$\left\{\begin{matrix} NMI\left(\Omega,\Pi\right)=\frac{2I\left(\Omega,\Pi\right)}{H\left(\Omega\right)+H\left(\Pi\right)} \\ I\left(\Omega,\Pi\right)=\sum_{i=1}^{M} \sum_{j=1}^{C} \frac{a_{ij}}{N}\log\frac{a_{ij}/N}{\left|t_{i}\right|\left|o_{j}\right|/N^{2}} \\ H\left(\Omega\right)=-\sum_{i=1}^{M} \frac{t_{i}}{N}\log\frac{t_{i}}{N} \\ H\left(\Pi\right)=-\sum_{j=1}^{C} \frac{o_{j}}{N}\log\frac{o_{j}}{N} \end{matrix}\right.$ (1)

where $I\left(\Pi,\Omega\right)$ is the mutual information and $H\left(\Pi\right)$ indicates the entropy, $\left|t_{i}\right|$ denotes the number of sequences in *t_i_*, $\left|o_{j}\right|$ is the number of sequences in *o_j_*, *a_ij_* represents the number of sequences from *s_i_* and categorized into *o_j_*. The range of NMI value is at [0, 1] and a larger NMI value implies better clustering accuracy.

**AMI calculation**

AMI is also based on mutual information and entropy, the AMI is calculated by:

$AMI\left(\Omega, \Pi\right)=\frac{I\left(\Omega, \Pi\right)-E\left[I\left(\Omega, \Pi\right)\right]}{\frac{1}{2}\left[H\left(\Omega\right)+H\left(\Pi\right)\right]-E\left[I\left(\Omega, \Pi\right)\right]}$ (2)

where $E\left[I\left(\Omega, \Pi\right)\right]$ is defined as:

$E\left[I\left(\Omega, \Pi\right)\right]=\sum_{i=1}^{M} \sum_{j=1}^{C} \sum_{k=\max\left(1,\left|t_{i}\right|+\left|o_{j}\right|-N\right)}^{\min\left(\left|t_{i}\right|, \left|o_{j}\right|\right)} \frac{k}{N}\log\left(\frac{N\times k}{\left|t_{i}\right|\times\left|o_{j}\right|}\right)\frac{\left|t_{i}\right|!\left|o_{j}\right|!\left(N-\left|t_{i}\right|\right)!\left(N-\left|o_{j}\right|\right)!}{N!k!\left(\left|t_{i}\right|-k\right)!\left(\left|o_{j}\right|-k\right)!\left(N-\left|t_{i}\right|-\left|o_{j}\right|+k\right)!}$ (3)

**ARI calculation**

ARI represents the number of pairwise sequences that are either in the same cluster or in different clusters in both partitions. The expected value between $\Omega$ and can be calculated by:

$\left\{\begin{matrix} ARI\left(\Omega, \prod\right)=\frac{\sum_{i}^{M} \sum_{j}^{C} \left(\begin{matrix} \left|a_{ij}\right| \\ 2 \end{matrix}\right)-E\left(\Omega, \prod\right)}{\frac{1}{2}\left[\sum_{i}^{M} \left(\begin{matrix} \left|t_{i}\right| \\ 2 \end{matrix}\right)+\sum_{j}^{C} \left(\begin{matrix} \left|o_{j}\right| \\ 2 \end{matrix}\right)\right]-E\left(\Omega, \prod\right)} \\ E\left(\Omega, \prod\right)=\frac{\sum_{i}^{M} \left(\begin{matrix} \left|t_{i}\right| \\ 2 \end{matrix}\right)\sum_{j}^{C} \left(\begin{matrix} \left|o_{j}\right| \\ 2 \end{matrix}\right)}{\left(\begin{matrix} n \\ 2 \end{matrix}\right)} \end{matrix}\right.$ (4)

**MCC calculation**

For a set of sequences that are clustered into OTUs by one algorithm at a distance threshold, sequence pairs could be classified as true positive (TP) samples, true negative (TN) samples, false positive (FP) samples, and false negative (FN) samples. A pair of sequences is classified as a TP sample if the pairwise sequence distance is smaller than the distance threshold and they are clustered into the same OTU; an FP sample is a pair of sequences that are clustered into the same OTU but their pairwise distance is larger than the threshold; a pair of sequences is classified as a TN sample if their pairwise distance is larger than the threshold and they are assigned to different OTUs; an FN sample is a pair of sequences that are clustered to different OTUs, but their pairwise distance is smaller than the threshold. The MCC is defined as:

$MCC=\frac{TP\times TN-FP\times FN}{\sqrt{\left(TP+FP\right)\left(TP+FN\right)\left(TN+FP\right)\left(TN+FN\right)}}$ (5)

**Supplementary Figures**

**Figure S1**. Processing procedures of obtaining ground-truth information for real-life datasets


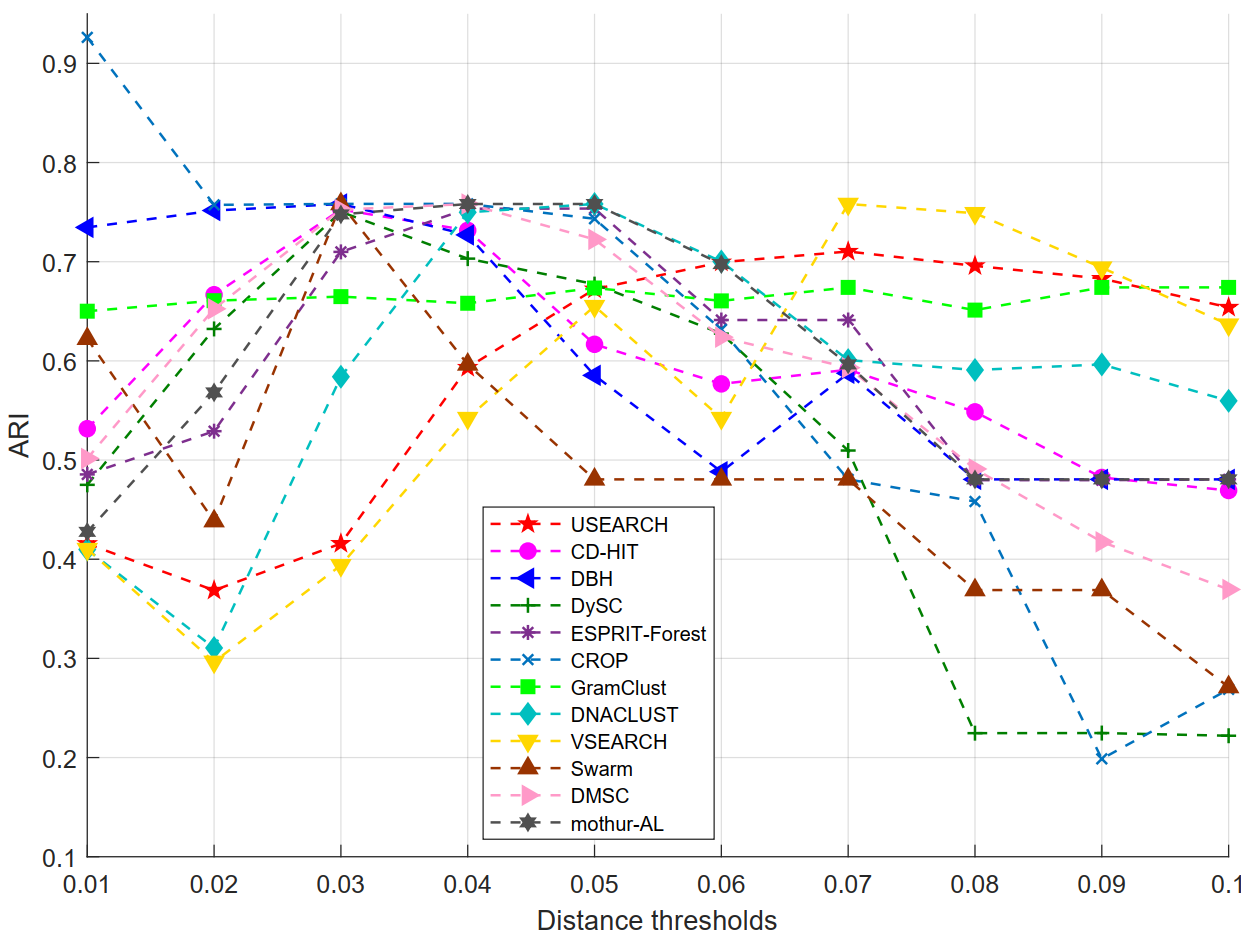


**Figure S2**. ARI values of different clustering methods on the simulated dataset.


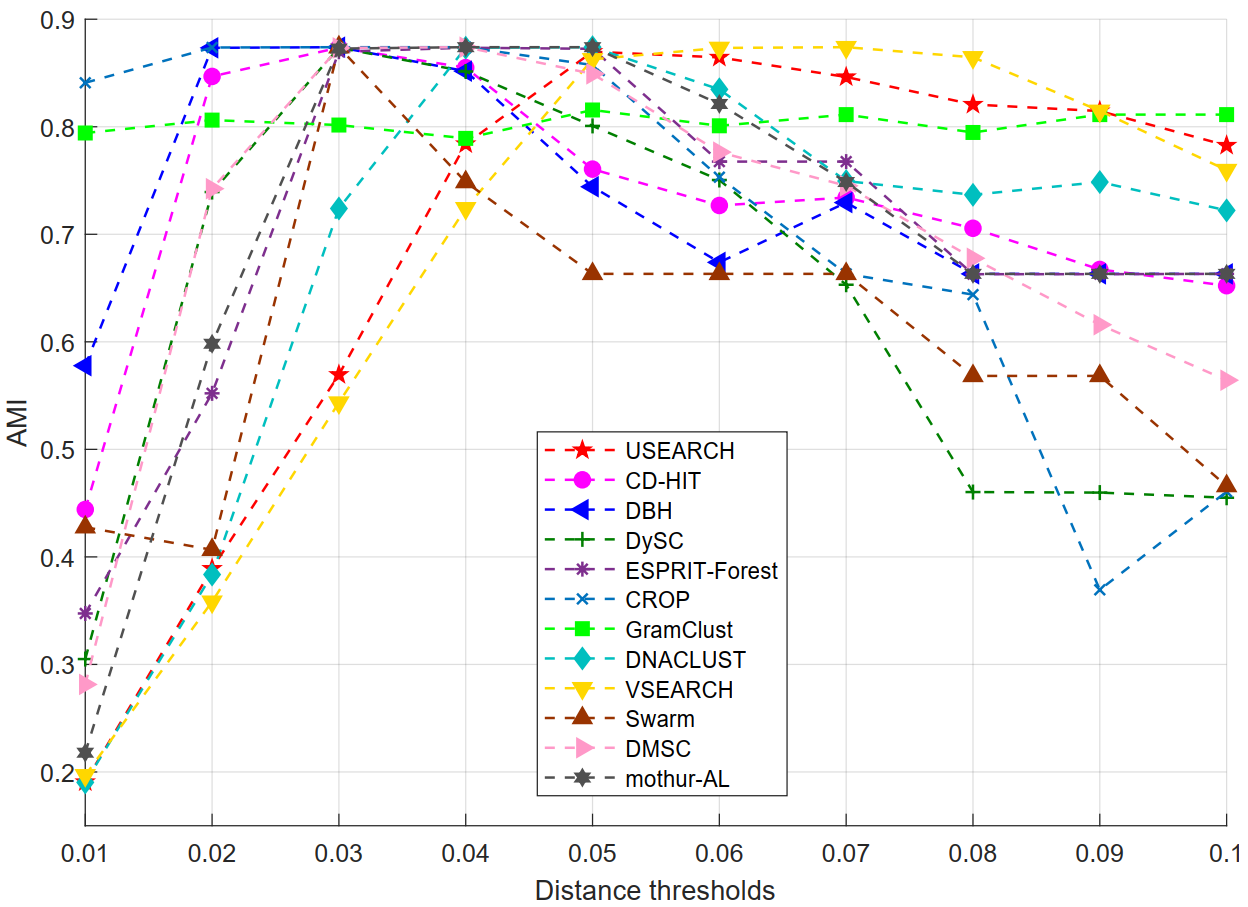


**Figure S3**. AMI values of different clustering methods on the simulated dataset.

**Figure S4**. OTUs number inferred with 8 clustering methods with different clustering thresholds on the V4 dataset.


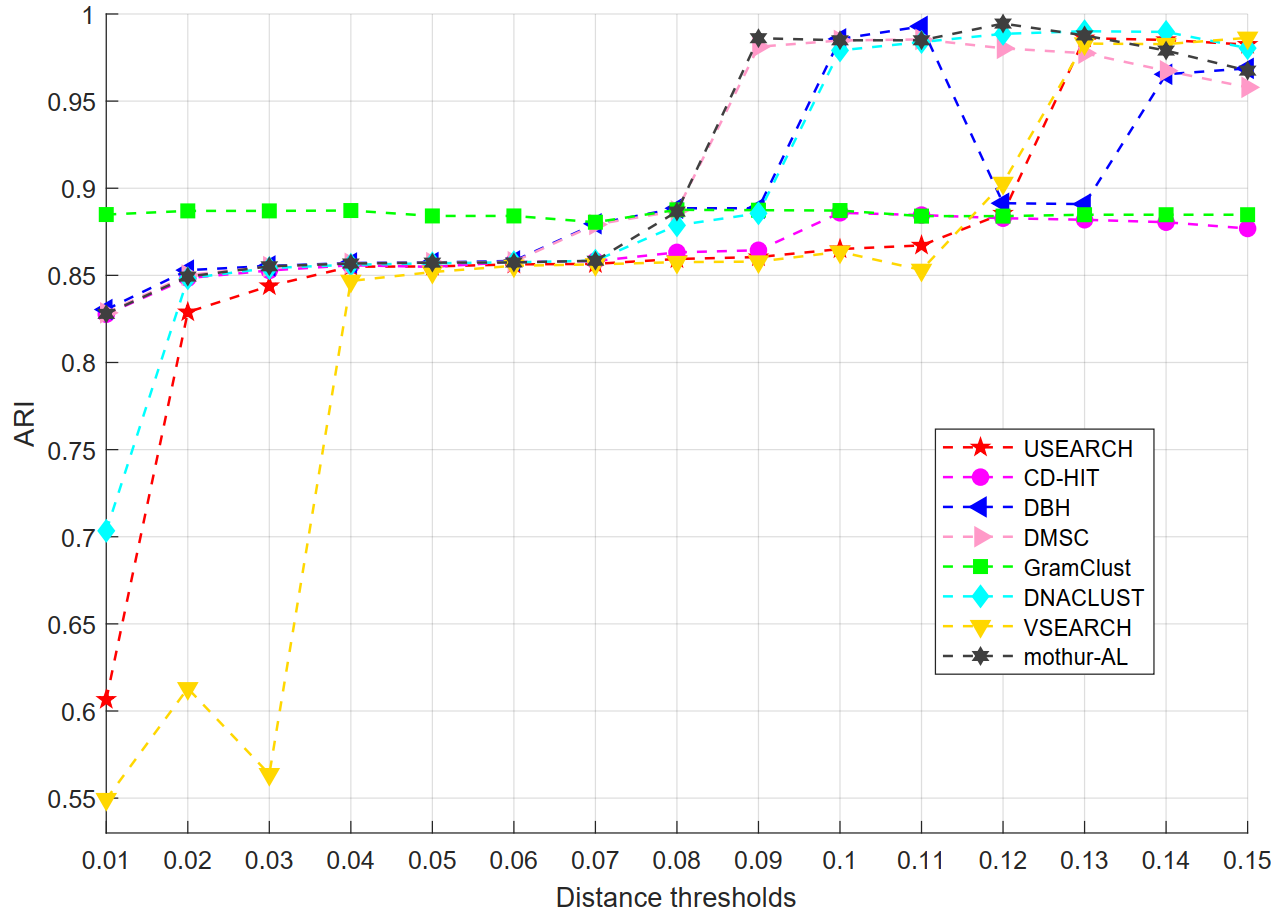


**Figure S5**. ARI values of 8 OTUs picking methods with different distance thresholds on the V4 dataset.


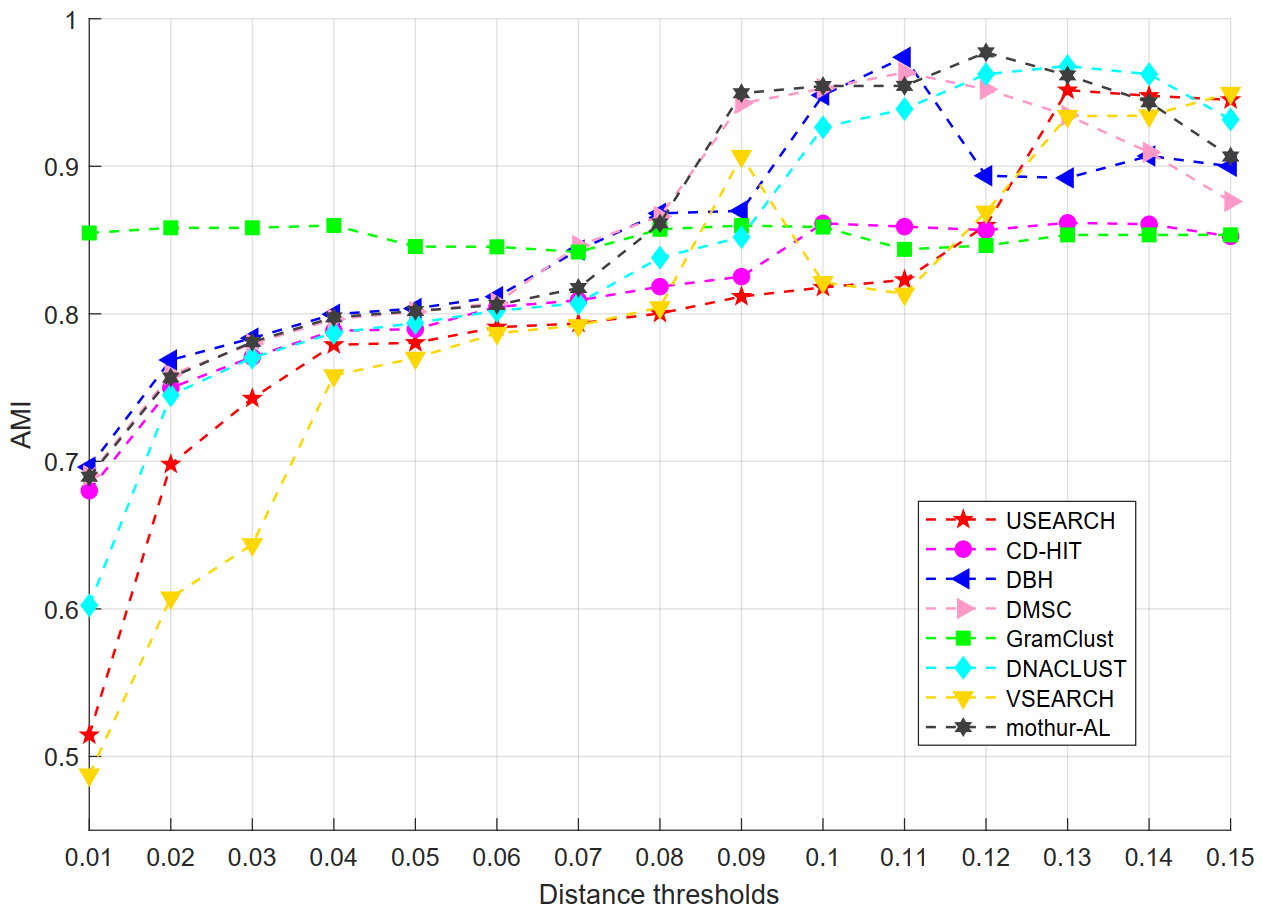


**Figure S6.** AMI values of 8 OTUs picking methods with different distance thresholds on the V4 dataset.


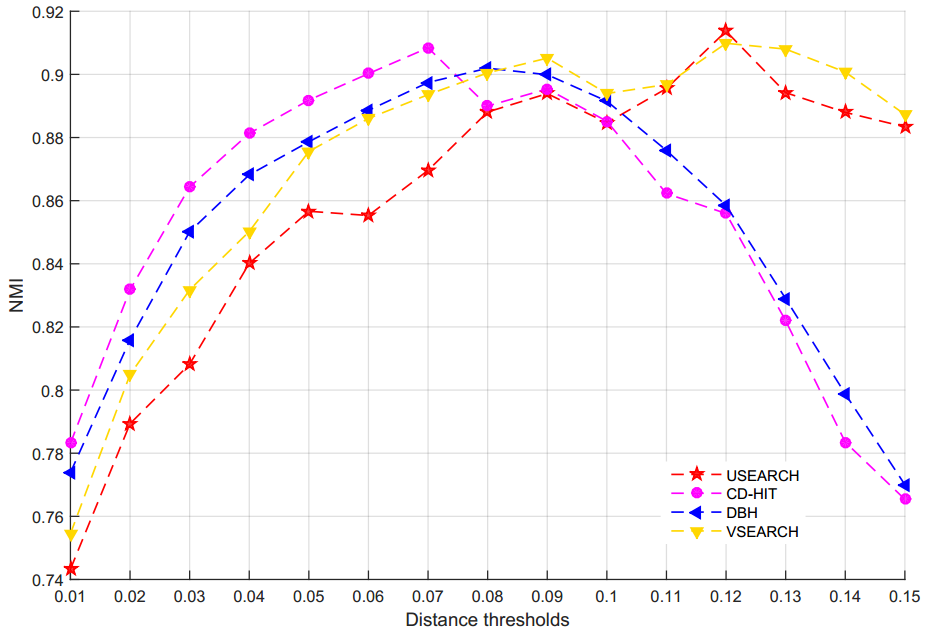


**Figure S7**. NMI values of 4 OTUs picking methods with different clustering thresholds on the full-length dataset.


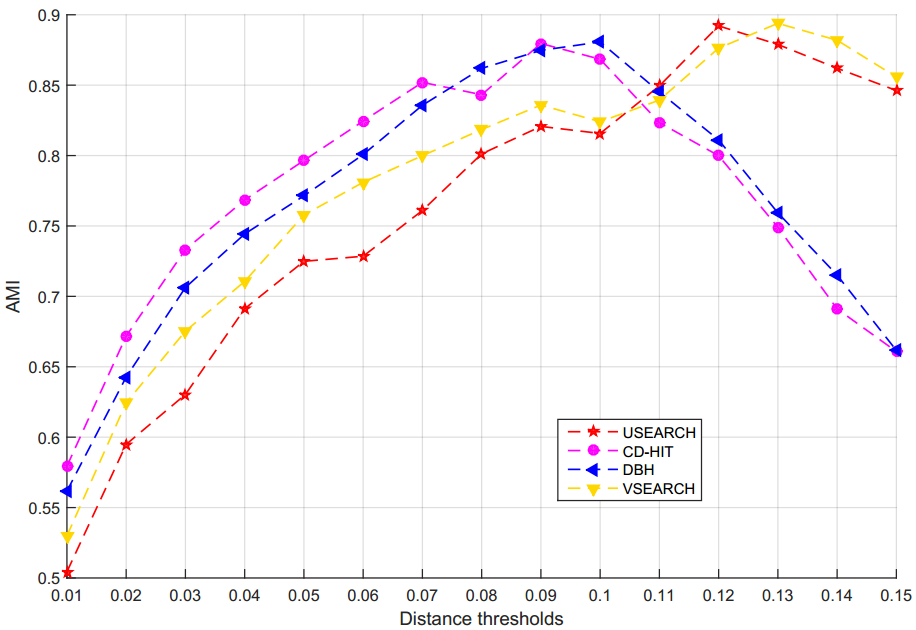


**Figure S8**. AMI values of 4 OTUs picking methods with different clustering thresholds on the full-length dataset.


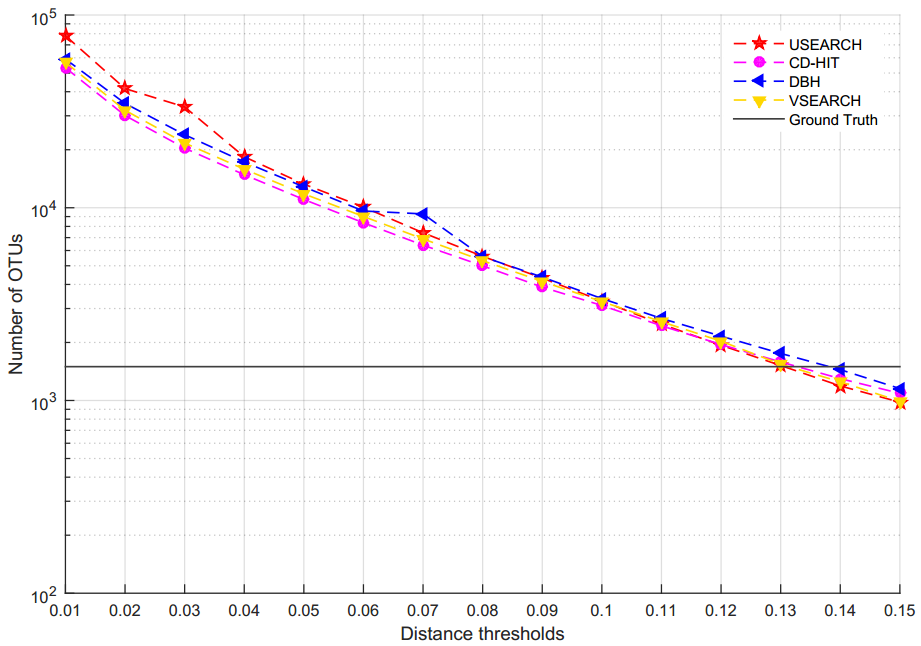


**Figure S9**. OTU number inferred with 4 OTUs picking methods with different clustering thresholds on the full-length dataset.


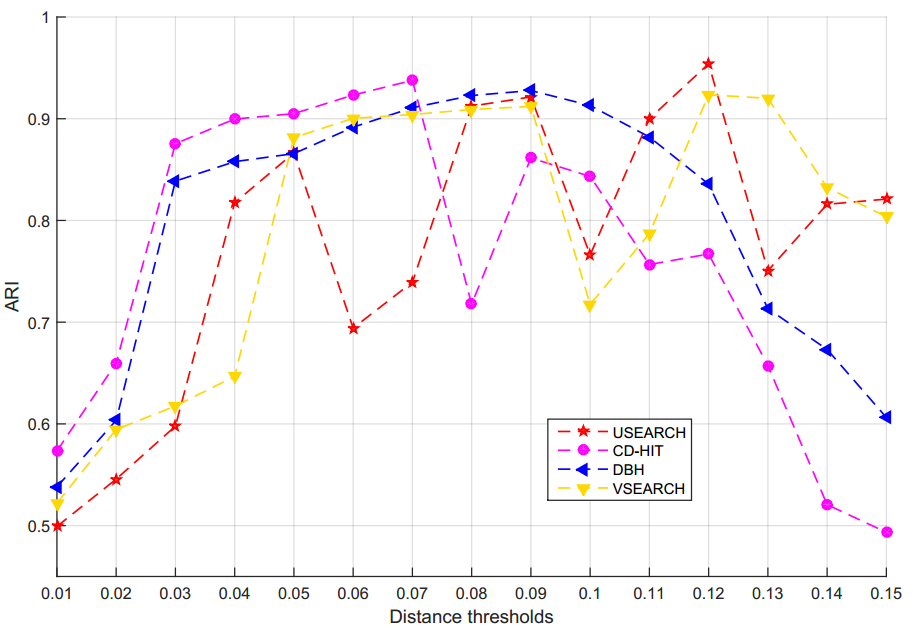


**Figure S10**. ARI values of 4 OTUs picking methods with different clustering thresholds on the full-length dataset.


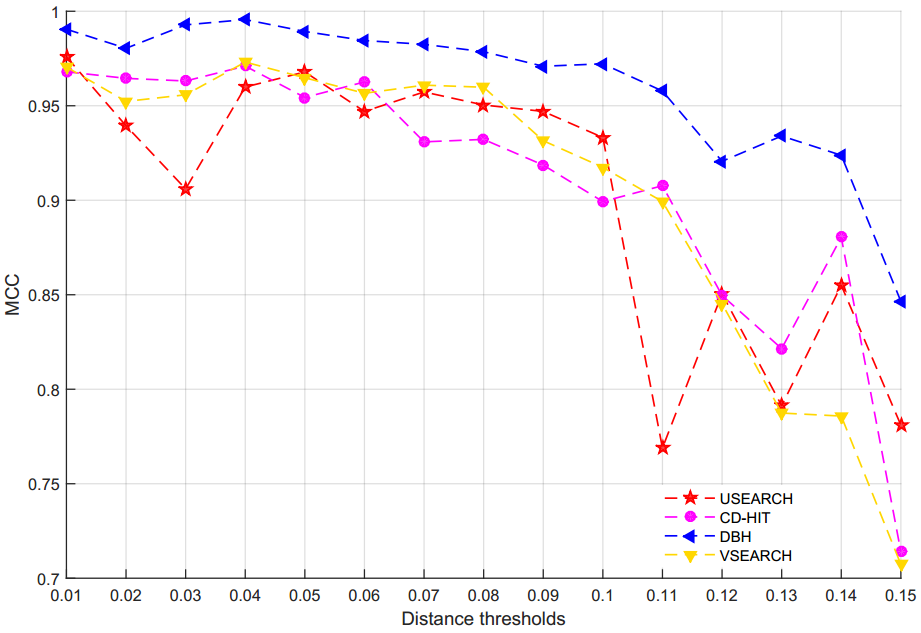


**Figure S11**. MCC values of 4 OTUs picking methods with different clustering thresholds on the full-length dataset.


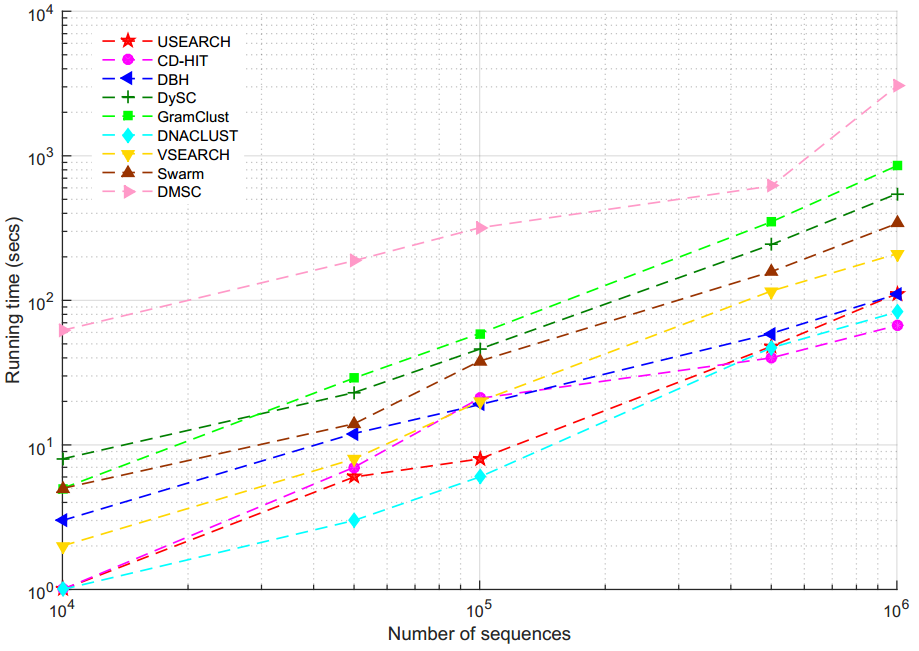

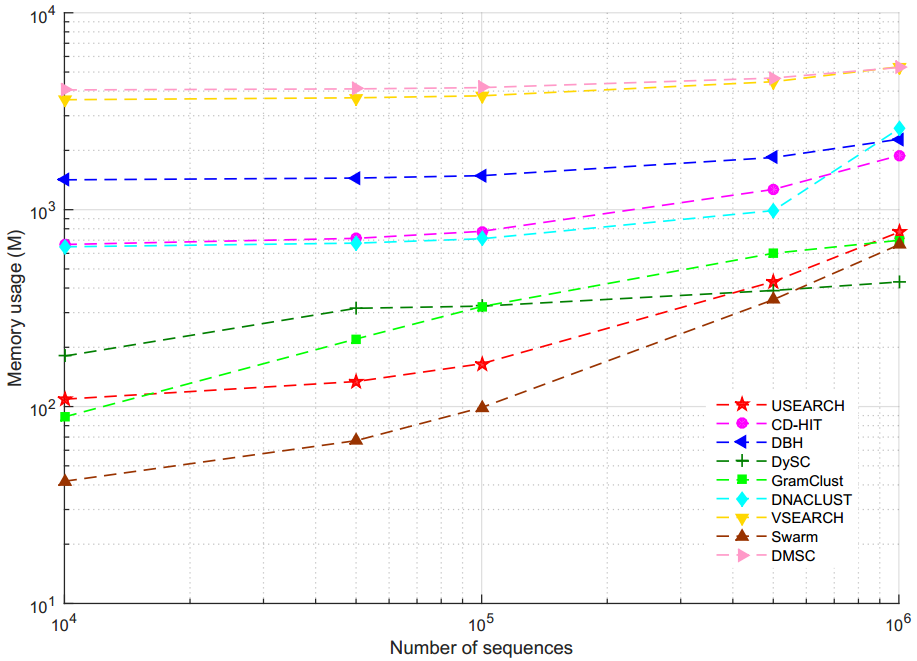


A. Running time B. Memory usage

**Figure S12**. Running time and memory usage of 9 heuristic clustering methods with different sequence sizes ranging from 10^4^ to 10^6^.

**Supplementary Tables**

**Table S1**. Running command lines of different methods for OTUs clustering

| Programs | Running command lines |
| --- | --- |
| ESPRIT-Forest | ESFclust seq.fa |
| DNACLUST | dnaclust seq.fa -l -t 16 -s 0.97 >dnaclust97.txt |
| GramCluster | GramCluster -i seq.fa -b 0.03 -o gramclust97.txt |
| VSEARCH | vsearch --cluster_fast seq.fa --uc vsearch97.txt --id 0.97 |
| CD-HIT | cd-hit -i seq.fasta -T 16 -M 0 -c 0.97 -o cdhit97 |
| DMSC | dmsc -i seq.fa -c 0.97 -o dmsc97.txt |
| CROP | CROP -i seq.fa -s -o crop97.txt |
| DySC | DySC-v0 -in seq.fasta -th 0.97 -out dysc97 |
| DBH | db -i seq.fasta -c 0.97 -o dbh97 -T 16 |
| swarm | vsearch --derep_fulllength seq.fa --sizein --sizeout --output uniq.fa --uc uc.txt |
|  | swarm -z -d 10 uniq.fa >swarm_d10.txt |
| USEARCH | usearch -sortbylength seq.fasta -fastaout sorted.fasta |
|  | usearch -cluster_fast sorted.fasta -id 0.97 -uc usearch97.uc |
| mothur-AL | unique.seqs(fasta=seq.fasta) |
|  | pairwise.seqs(fasta= unique.fa, cutoff=1) |
|  | cluster(column=unique.dist, name=seq.names, method=average, cutoff=0.99) |
|  | bin.seqs(list=seq.unique.an.list, fasta=seq.fasta) |

**Table S2**. The NMI, MCC values and OTUs number of twelve methods at different distance thresholds on simulated dataset.

| Distance | DMSC | | | USEARCH | | | CD-HIT | | | DBH | | | DySC | | | ESPRIT-Forest | | |
| --- | --- | --- | --- | --- | --- | --- | --- | --- | --- | --- | --- | --- | --- | --- | --- | --- | --- | --- |
|  | NMI | MCC | OTUs | NMI | MCC | OTUs | NMI | MCC | OTUs | NMI | MCC | OTUs | NMI | MCC | OTUs | NMI | MCC | OTUs |
| 0.01 | 0.9334 | 0.8808 | 99 | 0.7252 | **0.9369** | 528 | 0.5991 | 0.8295 | 927 | 0.7777 | 0.7080 | 439 | 0.6243 | 0.8940 | 941 | 0.5930 | 0.8903 | 1198 |
| 0.02 | **0.9503** | 0.9287 | 9 | 0.7759 | 0.8536 | 97 | 0.7160 | 0.8979 | 159 | 0.9161 | 0.8818 | 17 | 0.8140 | 0.9096 | 174 | 0.7815 | 0.9249 | 299 |
| 0.03 | 0.9434 | 0.9879 | 9 | 0.8524 | 0.7997 | 26 | 0.8555 | **0.9840** | 27 | **0.9293** | **0.9868** | 9 | **0.9252** | **0.9838** | 17 | 0.9114 | 0.9677 | 46 |
| 0.04 | 0.9307 | **0.9980** | 9 | 0.9000 | 0.8903 | 13 | 0.9267 | 0.9780 | 11 | 0.9106 | 0.9739 | 9 | 0.8950 | 0.9555 | 10 | 0.8967 | **0.9947** | 16 |
| 0.05 | 0.9223 | 0.9585 | 8 | **0.9107** | 0.9046 | 10 | **0.9334** | 0.8657 | 9 | 0.8433 | 0.8526 | 8 | 0.8641 | 0.9323 | 8 | **0.8979** | 0.9568 | 13 |
| 0.06 | 0.8958 | 0.9218 | 6 | 0.8814 | 0.8319 | 9 | 0.9009 | 0.8835 | 9 | 0.8298 | 0.8345 | 8 | 0.8497 | 0.8622 | 7 | 0.8529 | 0.8762 | 12 |
| 0.07 | 0.8698 | 0.9370 | 6 | 0.8552 | 0.7842 | 8 | 0.8518 | 0.9205 | 8 | 0.8054 | 0.8522 | 7 | 0.7700 | 0.7754 | 6 | 0.8512 | 0.8868 | 11 |
| 0.08 | 0.8353 | 0.9323 | 6 | 0.8169 | 0.7676 | 7 | 0.8244 | 0.9000 | 8 | 0.7986 | 0.9417 | 6 | 0.6874 | 0.5617 | 5 | 0.7956 | 0.9412 | 10 |
| 0.09 | 0.8261 | 0.9086 | 5 | 0.8174 | 0.7335 | 7 | 0.8052 | 0.9306 | 7 | 0.7866 | 0.9411 | 6 | 0.6365 | 0.5498 | 5 | 0.7445 | 0.9406 | 7 |
| 0.10 | 0.7966 | 0.9100 | 5 | 0.7898 | 0.6958 | 7 | 0.7652 | 0.9309 | 7 | 0.7686 | 0.9073 | 6 | 0.6107 | 0.5052 | 5 | 0.7245 | 0.9073 | 7 |

**Table S2-1**. The NMI, MCC values and OTUs number of twelve methods at different distance thresholds on simulated dataset.

| Distance | CROP | | | GramClust | | | DNACLUST | | | VSEARCH | | | Swarm | | | mothur-AL | | |
| --- | --- | --- | --- | --- | --- | --- | --- | --- | --- | --- | --- | --- | --- | --- | --- | --- | --- | --- |
|  | NMI | MCC | OTUs | NMI | MCC | OTUs | NMI | MCC | OTUs | NMI | MCC | OTUs | NMI | MCC | OTUs | NMI | MCC | OTUs |
| 0.01 | 0.9197 | 0.5842 | 158 | 0.8578 | 0.4045 | 9 | 0.5704 | 0.9340 | 1337 | 0.5725 | **0.9349** | 1291 | 0.6984 | 0.8045 | 769 | 0.5847 | 0.9549 | 1280 |
| 0.02 | 0.9320 | 0.8818 | 9 | 0.8691 | 0.8209 | 9 | 0.6228 | 0.8330 | 542 | 0.6082 | 0.8260 | 545 | 0.6540 | 0.9068 | 709 | 0.7485 | 0.9496 | 393 |
| 0.03 | **0.9334** | 0.9868 | 9 | 0.8679 | 0.903 | 9 | 0.8038 | 0.8969 | 143 | 0.7017 | 0.7898 | 202 | **0.9334** | **0.9868** | 9 | 0.9204 | 0.9879 | 31 |
| 0.04 | 0.9333 | **0.9980** | 9 | 0.8582 | 0.9061 | 9 | 0.9255 | **0.9921** | 15 | 0.7996 | 0.8601 | 62 | 0.8572 | 0.8354 | 7 | **0.9333** | **0.9980** | 9 |
| 0.05 | 0.9157 | 0.9485 | 9 | 0.8745 | **0.9106** | 9 | **0.9333** | 0.9601 | 9 | 0.8703 | 0.8932 | 24 | 0.7986 | 0.7496 | 6 | 0.9333 | 0.9980 | 9 |
| 0.06 | 0.8526 | 0.8477 | 7 | 0.8658 | 0.8453 | 9 | 0.8968 | 0.8767 | 9 | 0.9008 | 0.8310 | 15 | 0.7986 | 0.8345 | 6 | 0.9026 | 0.9136 | 8 |
| 0.07 | 0.7986 | 0.9066 | 6 | **0.8795** | 0.823 | 9 | 0.8255 | 0.8310 | 9 | **0.9334** | 0.7938 | 9 | 0.7986 | 0.9066 | 6 | 0.8572 | 0.9376 | 7 |
| 0.08 | 0.7784 | 0.9185 | 6 | 0.8573 | 0.783 | 9 | 0.8406 | 0.9002 | 8 | 0.9234 | 0.7627 | 9 | 0.7260 | 0.7982 | 5 | 0.7986 | 0.9417 | 6 |
| 0.09 | 0.6301 | 0.5078 | 5 | 0.8795 | 0.7494 | 9 | 0.8572 | 0.8620 | 7 | 0.8950 | 0.7659 | 8 | 0.7260 | 0.8464 | 5 | 0.7986 | 0.9417 | 6 |
| 0.10 | 0.5185 | 0.7673 | 4 | 0.8795 | 0.6863 | 9 | 0.8312 | 0.8059 | 7 | 0.8601 | 0.7467 | 7 | 0.6371 | 0.7696 | 4 | 0.7986 | 0.9417 | 6 |

mothur-AL software just outputs the clustering results at 0.03 distance threshold.

**Table S3.** Maximum ARI values of 12 OTUs picking methods on the simulated dataset.

|  | DMSC  (0.04) | USEARCH  (0.07) | DySC  (0.03) | ESPRIT-Forest (0.05) | CD-HIT (0.03) | CROP  (0.01) |
| --- | --- | --- | --- | --- | --- | --- |
| Max. ARI | 0.7582 | 0.7103 | 0.7503 | 0.7536 | 0.7526 | 0.9262 |
| OTUs number | 9 | 8 | 17 | 8 | 27 | 158 |
|  | VSEARCH (0.07) | DNACLUST  (0.05) | Swarm (*d*=15) | GramClust  (0.03) | DBH  (0.03) | mothur-AL  (0.04) |
| Max. ARI | 0.7582 | 0.7582 | 0.7582 | 0.6648 | 0.7582 | 0.7582 |
| OTUs number | 9 | 9 | 9 | 9 | 9 | 9 |

Note: The value in the bracket is the distance threshold where each method achieves its maximum ARI. For Swarm method, it is the value of parameter *d*.

**Table S4.** Maximum AMI values of 12 OTUs picking methods on the simulated dataset.

|  | DMSC  (0.04) | USEARCH  (0.05) | DySC  (0.04) | ESPRIT-Forest (0.04) | CD-HIT (0.03) | CROP  (0.05) |
| --- | --- | --- | --- | --- | --- | --- |
| Max. AMI | 0.8740 | 0.8698 | 0.8514 | 0.8735 | 0.8736 | 0.8574 |
| OTUs number | 9 | 10 | 10 | 10 | 27 | 9 |
|  | VSEARCH (0.07) | DNACLUST  (0.05) | Swarm (*d*=15) | GramClust  (0.09) | DBH  (0.03) | mothur-AL  (0.04) |
| Max. AMI | 0.8740 | 0.8740 | 0.8740 | 0.8113 | 0.8740 | 0.8740 |
| OTUs number | 9 | 9 | 9 | 9 | 9 | 9 |

Note: The value in the bracket is the distance threshold where each method achieves its maximum AMI. For Swarm method, it is the value of parameter *d*.

**Table S5**. The NMI, MCC values and OTUs number of eight methods at different distance thresholds on the V4 dataset.

| Distance | DMSC | | | USEARCH | | | CD-HIT | | | DBH | | |
| --- | --- | --- | --- | --- | --- | --- | --- | --- | --- | --- | --- | --- |
|  | NMI | MCC | OTUs | NMI | MCC | OTUs | NMI | MCC | OTUs | NMI | MCC | OTUs |
| 0.01 | 0.8187 | 0.8955 | 4378 | 0.6871 | 0.3681 | 11576 | 0.8117 | 0.4235 | 4839 | 0.8225 | 0.3844 | 4035 |
| 0.02 | 0.8632 | 0.9552 | 852 | 0.8229 | 0.8702 | 1044 | 0.8574 | 0.9189 | 806 | 0.8695 | 0.8946 | 613 |
| 0.03 | 0.8760 | 0.9765 | 326 | 0.8526 | 0.9205 | 448 | 0.8707 | 0.9614 | 353 | 0.8787 | 0.9587 | 240 |
| 0.04 | 0.8859 | 0.9891 | 169 | 0.8752 | **0.9797** | 197 | 0.8810 | 0.9814 | 181 | 0.8879 | 0.9806 | 142 |
| 0.05 | 0.8888 | **0.9913** | 133 | 0.8767 | 0.9768 | 159 | 0.8816 | **0.9876** | 145 | 0.8902 | **0.9875** | 126 |
| 0.06 | 0.8897 | 0.9902 | 115 | 0.8830 | 0.9736 | 119 | 0.8907 | 0.9828 | 120 | 0.8934 | 0.9761 | 99 |
| 0.07 | 0.9123 | 0.9773 | 95 | 0.8839 | 0.9587 | 108 | 0.8931 | 0.9731 | 102 | 0.9103 | 0.9157 | 90 |
| 0.08 | 0.9231 | 0.9297 | 78 | 0.8884 | 0.9015 | 95 | 0.8966 | 0.9194 | 88 | 0.9228 | 0.8811 | 77 |
| 0.09 | 0.9612 | 0.9078 | 67 | 0.8949 | 0.8454 | 82 | 0.8979 | 0.8812 | 80 | 0.9235 | 0.8881 | 72 |
| 0.10 | 0.9651 | 0.9355 | 61 | 0.8969 | 0.8170 | 70 | **0.9179** | 0.8817 | 67 | **0.9618** | 0.9406 | 60 |
| 0.11 | **0.9681** | 0.9731 | 58 | 0.8982 | 0.7829 | 62 | 0.9133 | 0.8457 | 61 | 0.9604 | 0.9735 | 52 |
| 0.12 | 0.9656 | 0.9563 | 48 | 0.9175 | 0.8027 | 56 | 0.9078 | 0.9210 | 55 | 0.9214 | 0.9302 | 47 |
| 0.13 | 0.9560 | 0.9252 | 44 | 0.9602 | 0.8780 | 49 | 0.9033 | 0.8141 | 49 | 0.9201 | 0.9216 | 43 |
| 0.14 | 0.9448 | 0.9012 | 40 | **0.9629** | 0.8367 | 45 | 0.8983 | 0.7572 | 44 | 0.9430 | 0.8875 | 38 |
| 0.15 | 0.9239 | 0.9162 | 35 | 0.9570 | 0.8108 | 43 | 0.8817 | 0.7965 | 43 | 0.9331 | 0.8872 | 37 |

**Table S5-1**. The NMI, MCC values and OTUs number of eight methods at different distance thresholds on the V4 dataset.

| Distance | GramClust | | | DNACLUST | | | VSEARCH | | | mothur-AL | | |
| --- | --- | --- | --- | --- | --- | --- | --- | --- | --- | --- | --- | --- |
|  | NMI | MCC | OTUs | NMI | MCC | OTUs | NMI | MCC | OTUs | NMI | MCC | OTUs |
| 0.01 | 0.9130 | 0.2174 | 100 | 0.7558 | 0.9533 | 6777 | 0.6609 | 0.6682 | 5860 | 0.8180 | 0.4943 | 3936 |
| 0.02 | 0.9174 | 0.7353 | 105 | 0.8542 | 0.9474 | 1003 | 0.7569 | 0.6675 | 974 | 0.8619 | 0.9364 | 762 |
| 0.03 | 0.9174 | 0.8029 | 105 | 0.8705 | 0.9728 | 451 | 0.7837 | 0.8198 | 417 | 0.8771 | 0.9674 | 240 |
| 0.04 | **0.9188** | 0.8313 | 109 | 0.8804 | 0.9867 | 192 | 0.8626 | 0.9320 | 191 | 0.8866 | 0.9878 | 146 |
| 0.05 | 0.9104 | 0.8380 | 108 | 0.8847 | **0.9884** | 154 | 0.8701 | 0.9660 | 152 | 0.8892 | 0.9894 | 118 |
| 0.06 | 0.9105 | 0.8457 | 106 | 0.8893 | 0.9865 | 125 | 0.8802 | **0.9746** | 122 | 0.8916 | **0.9904** | 95 |
| 0.07 | 0.9075 | 0.8574 | 109 | 0.8926 | 0.9753 | 107 | 0.8830 | 0.9607 | 106 | 0.8954 | 0.9786 | 80 |
| 0.08 | 0.9164 | **0.9083** | 108 | 0.9104 | 0.9420 | 91 | 0.8902 | 0.9102 | 91 | 0.9200 | 0.9322 | 67 |
| 0.09 | 0.9193 | 0.8748 | 107 | 0.9167 | 0.9187 | 82 | 0.8918 | 0.8608 | 83 | 0.9679 | 0.9354 | 60 |
| 0.10 | 0.9177 | 0.8411 | 112 | 0.9552 | 0.9220 | 64 | 0.8968 | 0.8141 | 68 | 0.9658 | 0.9420 | 55 |
| 0.11 | 0.9091 | 0.8192 | 111 | 0.9591 | 0.9393 | 63 | 0.8919 | 0.7802 | 64 | 0.9658 | 0.9757 | 52 |
| 0.12 | 0.9102 | 0.7976 | 103 | 0.9681 | 0.943 | 58 | 0.9242 | 0.7974 | 57 | **0.9809** | 0.9608 | 44 |
| 0.13 | 0.9139 | 0.7534 | 102 | 0.9689 | 0.9342 | 54 | 0.9589 | 0.8779 | 52 | 0.9729 | 0.9388 | 42 |
| 0.14 | 0.9139 | 0.7161 | 102 | **0.9705** | 0.9165 | 47 | 0.9560 | 0.8348 | 48 | 0.9634 | 0.9217 | 40 |
| 0.15 | 0.9139 | 0.6687 | 102 | 0.9496 | 0.8912 | 43 | **0.9624** | 0.8025 | 41 | 0.9510 | 0.9194 | 35 |

mothur-AL software just outputs the clustering results at 0.01, 0.02 and 0.03 distance thresholds.

**Table S6**. The ARI and AMI values of eight methods at different distance thresholds on the V4 dataset.

|  | DMSC | | USEARCH | | CD-HIT | | DBH | | GramClust | | DNACLUST | | VSEARCH | | mothur-AL | |
| --- | --- | --- | --- | --- | --- | --- | --- | --- | --- | --- | --- | --- | --- | --- | --- | --- |
|  | ARI | AMI | ARI | AMI | ARI | AMI | ARI | AMI | ARI | AMI | ARI | AMI | ARI | AMI | ARI | AMI |
| 0.01 | 0.8285 | 0.6905 | 0.6065 | 0.5145 | 0.8277 | 0.6801 | 0.8304 | 0.6961 | 0.8849 | 0.8548 | 0.7034 | 0.6024 | 0.5494 | 0.4876 | 0.8279 | 0.6896 |
| 0.02 | 0.8502 | 0.7587 | 0.8289 | 0.698 | 0.8484 | 0.7498 | 0.8530 | 0.7687 | 0.8870 | 0.8583 | 0.8483 | 0.7448 | 0.6130 | 0.6076 | 0.8493 | 0.7568 |
| 0.03 | 0.8550 | 0.7797 | 0.8439 | 0.7427 | 0.8528 | 0.7707 | 0.8554 | 0.7835 | 0.8870 | 0.8583 | 0.8542 | 0.7703 | 0.5637 | 0.6437 | 0.8552 | 0.7809 |
| 0.04 | 0.8569 | 0.7964 | 0.8550 | 0.7790 | 0.8558 | 0.7885 | 0.8571 | 0.7997 | 0.8872 | 0.8599 | 0.8561 | 0.787 | 0.8468 | 0.7582 | 0.8568 | 0.7976 |
| 0.05 | 0.8573 | 0.8014 | 0.8551 | 0.7804 | 0.8549 | 0.7896 | 0.8574 | 0.8036 | 0.8841 | 0.8456 | 0.8569 | 0.7938 | 0.8519 | 0.7701 | 0..8573 | 0.8019 |
| 0.06 | 0.8578 | 0.8074 | 0.8563 | 0.7909 | 0.8577 | 0.8047 | 0.8583 | 0.8116 | 0.8841 | 0.8454 | 0.8575 | 0.802 | 0.8555 | 0.7869 | 0.8574 | 0.8060 |
| 0.07 | 0.8792 | 0.8463 | 0.8566 | 0.7935 | 0.8582 | 0.8091 | 0.8796 | 0.8432 | 0.8805 | 0.8419 | 0.8584 | 0.8072 | 0.8563 | 0.7921 | 0.8584 | 0.8175 |
| 0.08 | 0.8870 | 0.8660 | 0.8594 | 0.8003 | 0.8632 | 0.8184 | 0.8885 | 0.8681 | **0.8876** | 0.8573 | 0.8787 | 0.8381 | 0.8576 | 0.8043 | 0.8866 | 0.8613 |
| 0.09 | 0.9812 | 0.9427 | 0.8605 | 0.8117 | 0.8644 | 0.8252 | 0.8885 | 0.8698 | 0.8874 | **0.8598** | 0.8856 | 0.852 | 0.8579 | 0.9069 | 0.9861 | 0.9495 |
| 0.10 | 0.9848 | 0.9527 | 0.8651 | 0.8179 | **0.8859** | 0.8613 | 0.9857 | 0.9482 | 0.8872 | 0.8588 | 0.9791 | 0.9264 | 0.8635 | 0.8214 | 0.9849 | 0.9544 |
| 0.11 | **0.9854** | **0.9636** | 0.8672 | 0.8230 | 0.8846 | 0.8591 | 0.9929 | 0.9739 | 0.8840 | 0.8437 | 0.9839 | 0.9388 | 0.8531 | 0.8135 | 0.9849 | 0.9545 |
| 0.12 | 0.9803 | 0.9521 | 0.8855 | 0.8599 | 0.8828 | 0.8567 | 0.8914 | 0.8936 | 0.8840 | 0.8463 | 0.9886 | 0.9624 | 0.9030 | 0.8691 | **0.9945** | **0.9772** |
| 0.13 | 0.9775 | 0.9345 | **0.9861** | **0.9516** | 0.8819 | **0.8617** | 0.8909 | 0.8921 | 0.8848 | 0.8535 | 0.9901 | **0.9679** | 0.9830 | 0.9340 | 0.9877 | 0.9616 |
| 0.14 | 0.9674 | 0.9095 | 0.9851 | 0.9478 | 0.8805 | 0.8607 | 0.9654 | **0.9070** | 0.8848 | 0.8535 | **0.9897** | 0.9624 | 0.9827 | 0.9343 | 0.9790 | 0.9434 |
| 0.15 | 0.9579 | 0.8762 | 0.9824 | 0.9450 | 0.8769 | 0.8525 | **0.9688** | 0.9000 | 0.8848 | 0.8535 | 0.9804 | 0.9317 | **0.9863** | **0.9496** | 0.9675 | 0.9066 |

mothur-AL software just outputs the clustering results at 0.01, 0.02 and 0.03 distance thresholds.

**Table S7**. The NMI, MCC, ARI, AMI values and OTUs number of four methods at different distance thresholds on the full-length dataset.

| Distance thresholds | | 0.01 | 0.02 | 0.03 | 0.04 | 0.05 | 0.06 | 0.07 | 0.08 | 0.09 | 0.10 | 0.11 | 0.12 | 0.13 | 0.14 | 0.15 |
| --- | --- | --- | --- | --- | --- | --- | --- | --- | --- | --- | --- | --- | --- | --- | --- | --- |
| USEARCH | NMI | 0.7432 | 0.7893 | 0.8082 | 0.8401 | 0.8566 | 0.8552 | 0.8695 | 0.8880 | 0.8939 | 0.8846 | 0.8955 | 0.9136 | 0.8942 | 0.8879 | 0.8833 |
|  | AMI | 0.5044 | 0.5949 | 0.6303 | 0.6912 | 0.7249 | 0.7285 | 0.7610 | 0.8012 | 0.8207 | 0.8157 | 0.8495 | 0.8921 | 0.8789 | 0.8620 | 0.8462 |
|  | ARI | 0.4992 | 0.5457 | 0.5978 | 0.8174 | 0.8665 | 0.6942 | 0.7393 | 0.9124 | 0.9214 | 0.7661 | 0.9000 | 0.954 | 0.7506 | 0.8159 | 0.8210 |
|  | MCC | 0.9759 | 0.9394 | 0.906 | 0.9599 | 0.9679 | 0.9470 | 0.9573 | 0.9504 | 0.947 | 0.9332 | 0.7689 | 0.8505 | 0.7913 | 0.855 | 0.7810 |
|  | OTUs | 78081 | 41528 | 33352 | 18391 | 13261 | 10078 | 7416 | 5598 | 4313 | 3282 | 2490 | 1935 | 1519 | 1190 | 980 |
| CD-HIT | NMI | 0.7831 | 0.8317 | 0.8643 | 0.8813 | 0.8918 | 0.9002 | 0.9084 | 0.8899 | 0.8954 | 0.8851 | 0.8622 | 0.8559 | 0.8219 | 0.7834 | 0.7653 |
|  | AMI | 0.5794 | 0.6718 | 0.7332 | 0.7685 | 0.7965 | 0.8245 | 0.852 | 0.8432 | 0.8796 | 0.8684 | 0.8234 | 0.7999 | 0.7491 | 0.6911 | 0.6608 |
|  | ARI | 0.5734 | 0.6598 | 0.8755 | 0.8997 | 0.905 | 0.9233 | 0.9377 | 0.7177 | 0.8614 | 0.8433 | 0.7567 | 0.767 | 0.6573 | 0.5206 | 0.4934 |
|  | MCC | 0.968 | 0.9645 | 0.9631 | 0.971 | 0.9544 | 0.9626 | 0.9309 | 0.9322 | 0.9186 | 0.8993 | 0.9077 | 0.8496 | 0.8215 | 0.8804 | 0.714 |
|  | OTUs | 53273 | 30099 | 20380 | 14839 | 11038 | 8358 | 6397 | 4995 | 3875 | 3111 | 2442 | 1950 | 1585 | 1298 | 1089 |
| DBH | NMI | 0.7738 | 0.8151 | 0.8501 | 0.8681 | 0.8786 | 0.8886 | 0.8974 | 0.9019 | 0.8999 | 0.8915 | 0.8759 | 0.8584 | 0.8289 | 0.79880 | 0.7700 |
|  | AMI | 0.5617 | 0.6427 | 0.7063 | 0.7443 | 0.7714 | 0.8007 | 0.8357 | 0.8619 | 0.8747 | 0.8807 | 0.8452 | 0.8111 | 0.7593 | 0.7151 | 0.6616 |
|  | ARI | 0.5381 | 0.6035 | 0.8387 | 0.8578 | 0.8657 | 0.8917 | 0.9108 | 0.9228 | 0.9277 | 0.913 | 0.882 | 0.8357 | 0.7133 | 0.6732 | 0.6063 |
|  | MCC | 0.9905 | 0.9804 | 0.9928 | 0.9956 | 0.9893 | 0.9845 | 0.9825 | 0.9786 | 0.9709 | 0.9722 | 0.9578 | 0.9206 | 0.934 | 0.9238 | 0.8465 |
|  | OTUs | 59067 | 34778 | 23927 | 17279 | 12837 | 9619 | 9291 | 5575 | 4367 | 3380 | 2666 | 2157 | 1755 | 1445 | 1153 |
| VSEARCH | NMI | 0.7544 | 0.8049 | 0.8317 | 0.8502 | 0.8755 | 0.8860 | 0.8935 | 0.9004 | 0.9051 | 0.8939 | 0.8967 | 0.9098 | 0.9080 | 0.9007 | 0.8873 |
|  | AMI | 0.5299 | 0.6244 | 0.6753 | 0.7106 | 0.7578 | 0.7810 | 0.7999 | 0.8185 | 0.8359 | 0.824 | 0.8394 | 0.8763 | 0.8939 | 0.8819 | 0.8558 |
|  | ARI | 0.5221 | 0.5945 | 0.6176 | 0.6466 | 0.8815 | 0.9004 | 0.9041 | 0.9091 | 0.9121 | 0.7175 | 0.787 | 0.9235 | 0.9201 | 0.8322 | 0.8036 |
|  | MCC | 0.9705 | 0.9523 | 0.9558 | 0.9731 | 0.9648 | 0.9566 | 0.9609 | 0.9598 | 0.9315 | 0.9174 | 0.8993 | 0.8453 | 0.7874 | 0.7858 | 0.7073 |
|  | OTUs | 56800 | 32050 | 21665 | 15854 | 11804 | 9012 | 6893 | 5314 | 4146 | 3260 | 2563 | 2031 | 1542 | 1250 | 990 |
